# Supplementary figures and images for: Taxifolin protects rat against myocardial ischemia/reperfusion injury by modulating the mitochondrial apoptosis pathway
Source: PeerJ. 2019 Jan 31;7:e6383. doi: 10.7717/peerj.6383 (PMC6360081; doi:10.7717/peerj.6383)

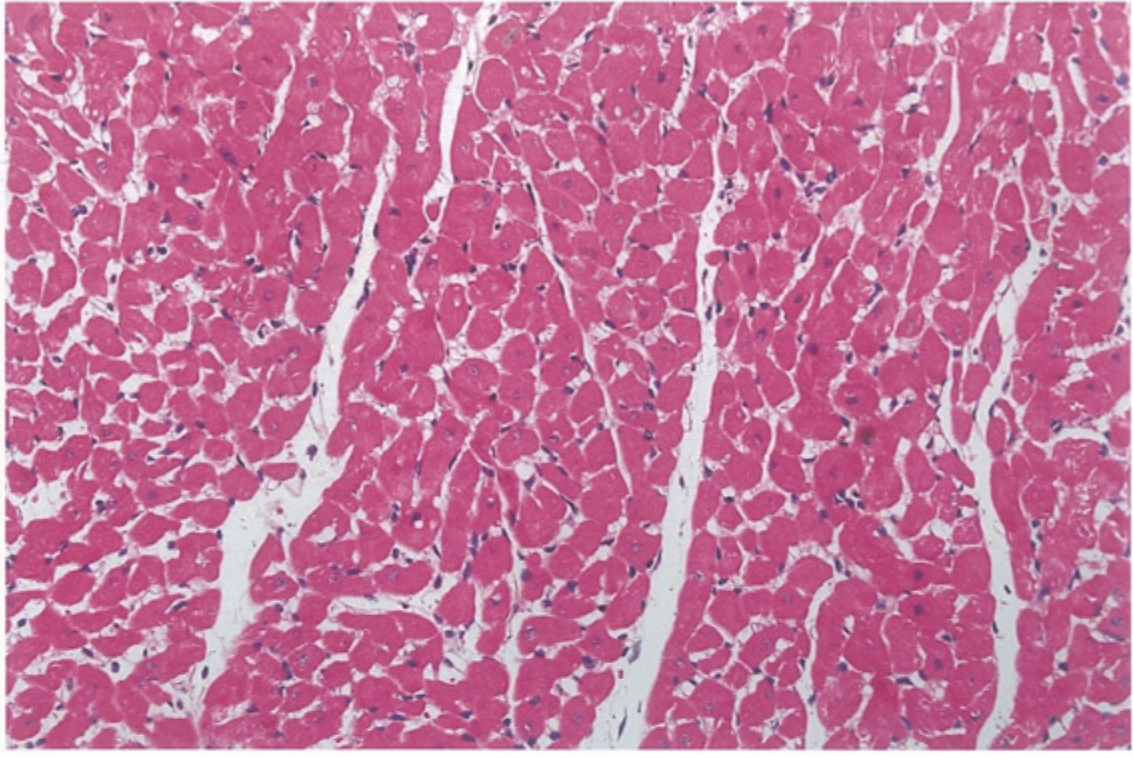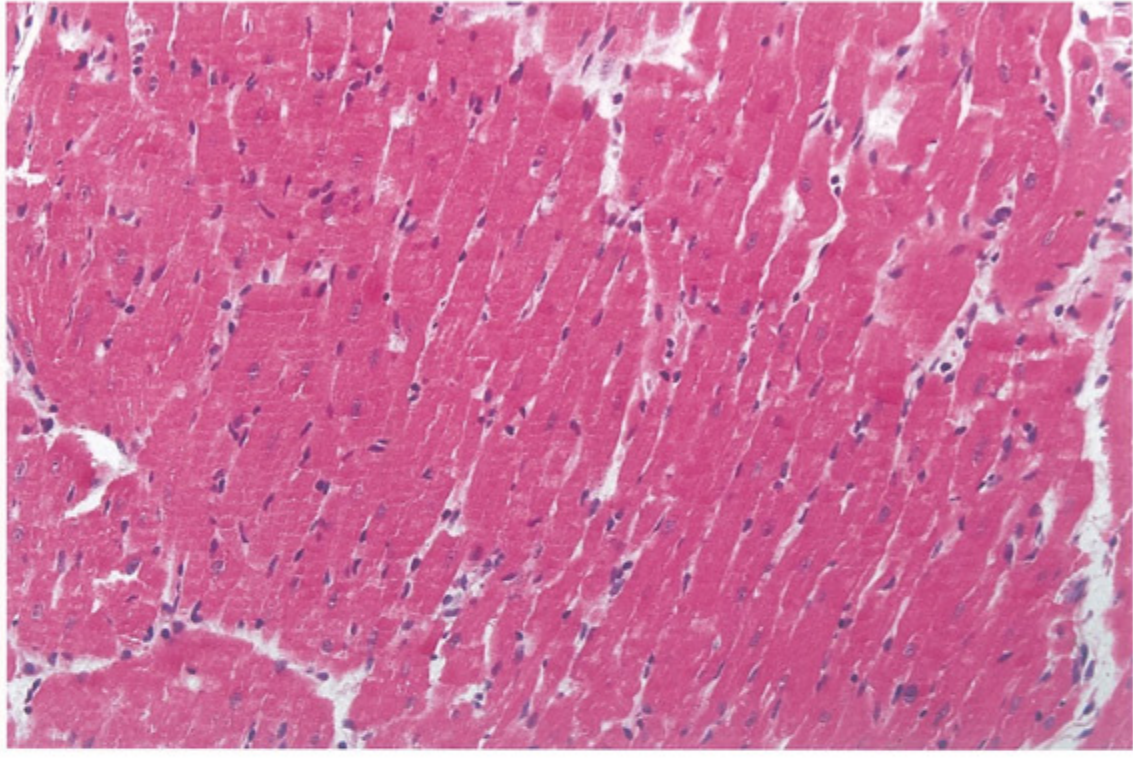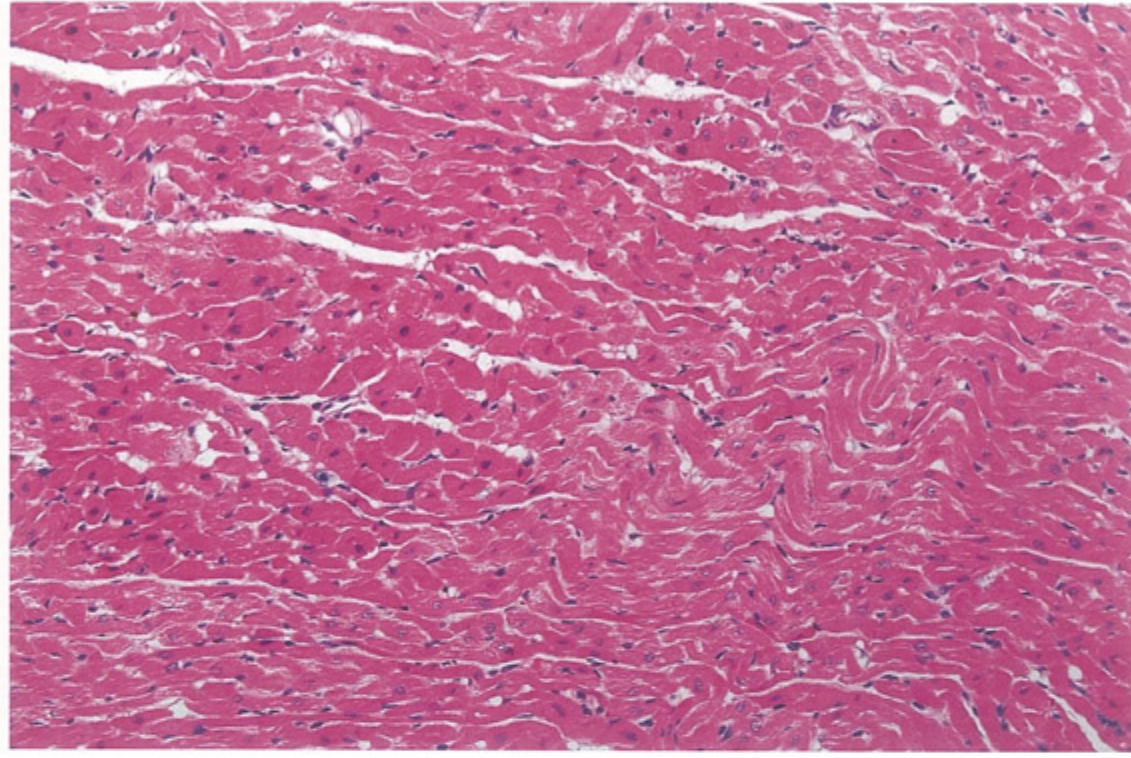

**Control**

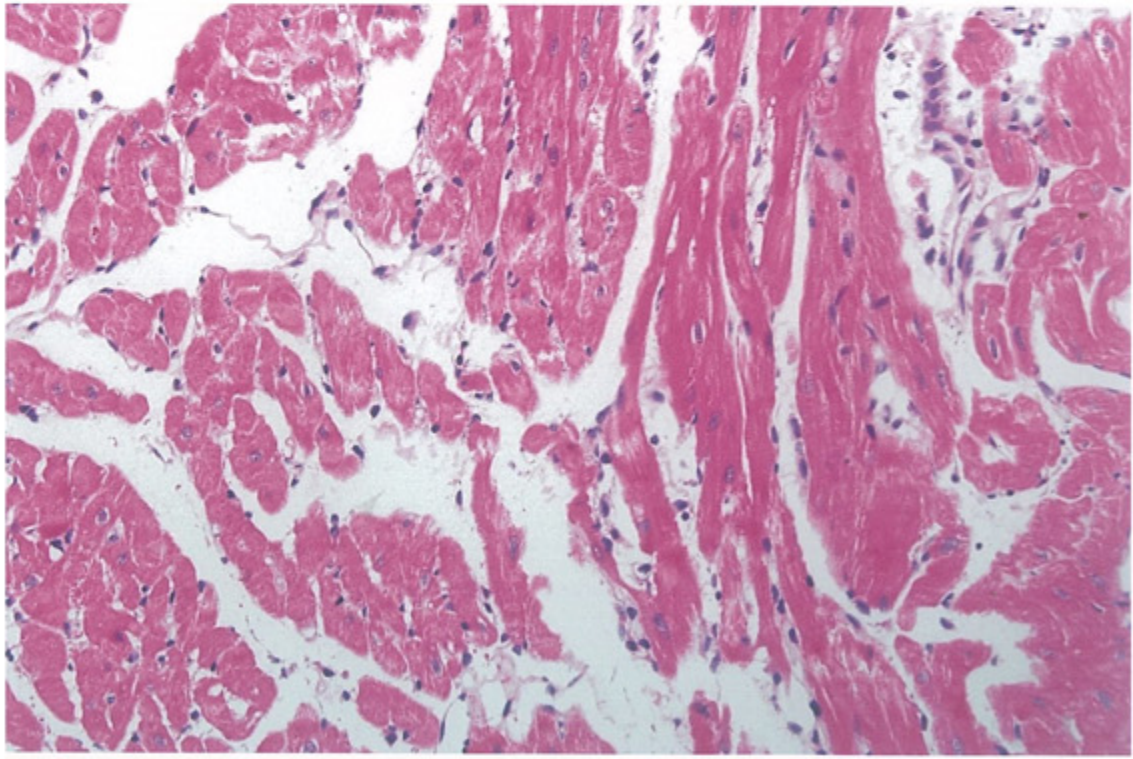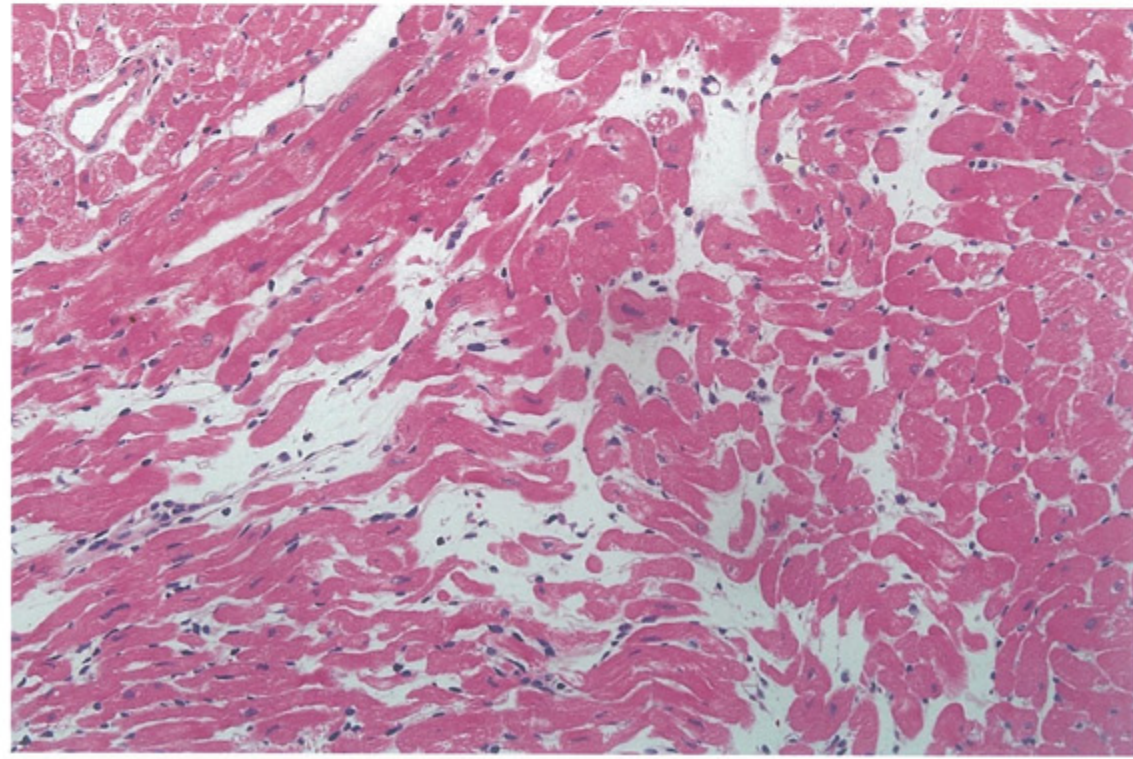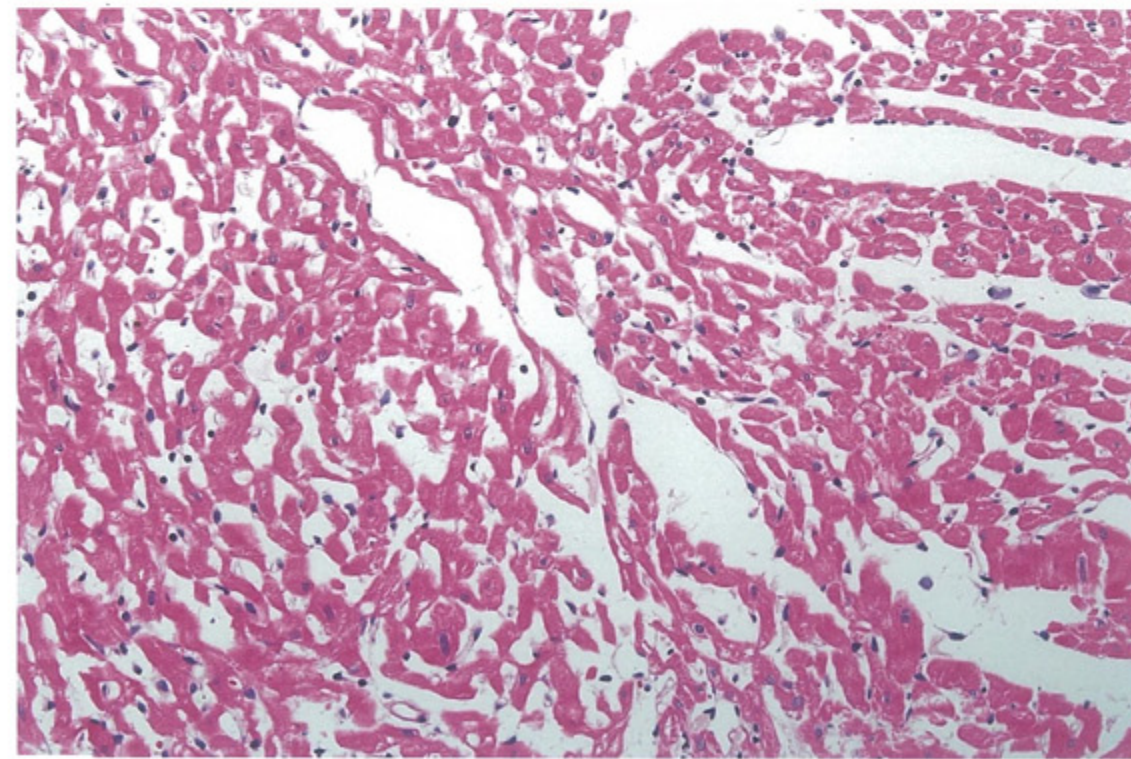

**I/R**

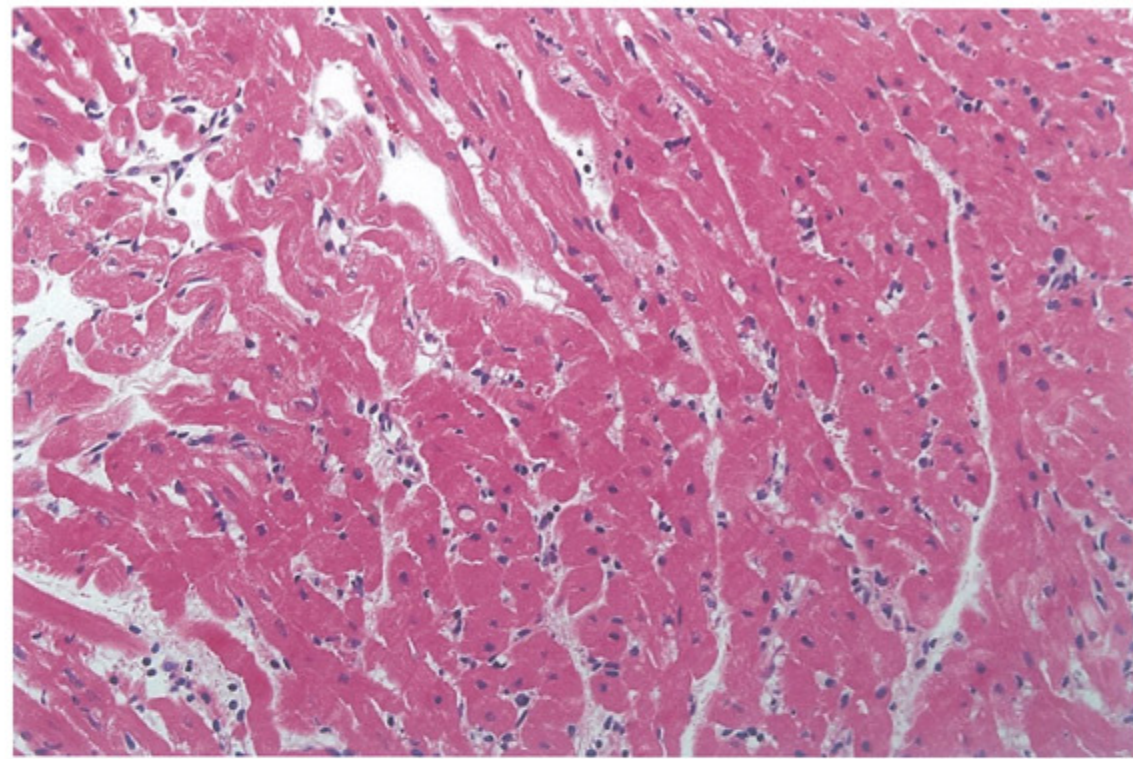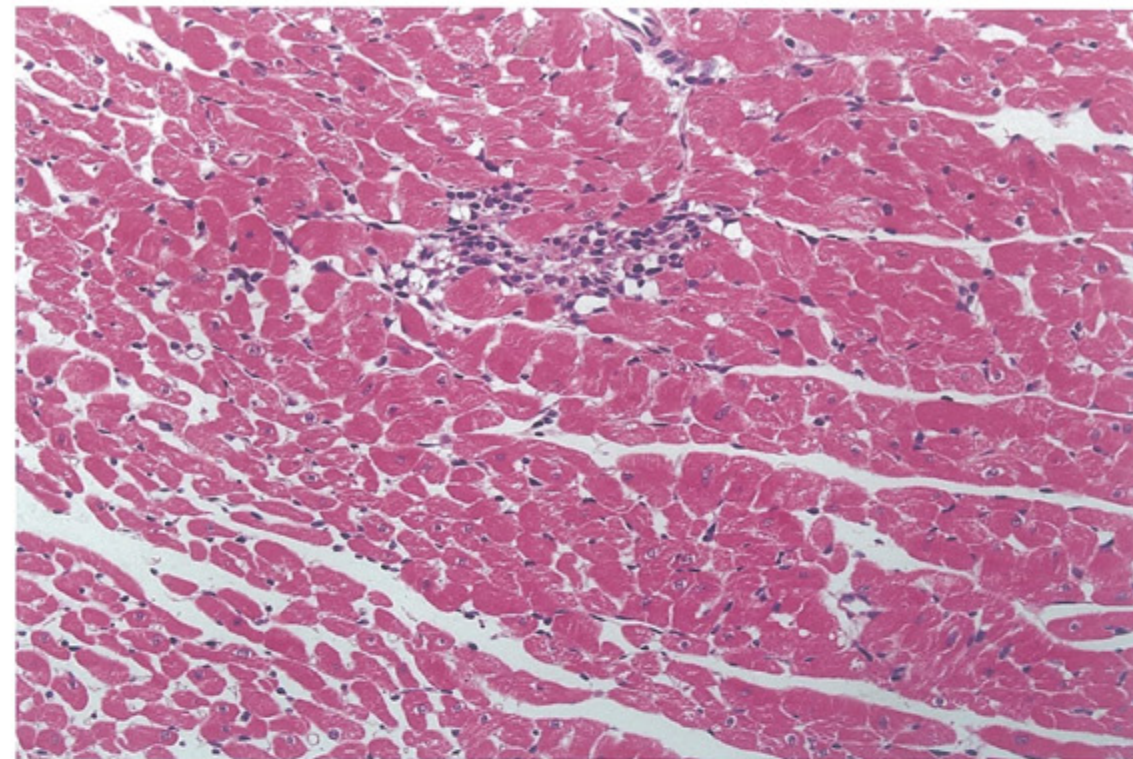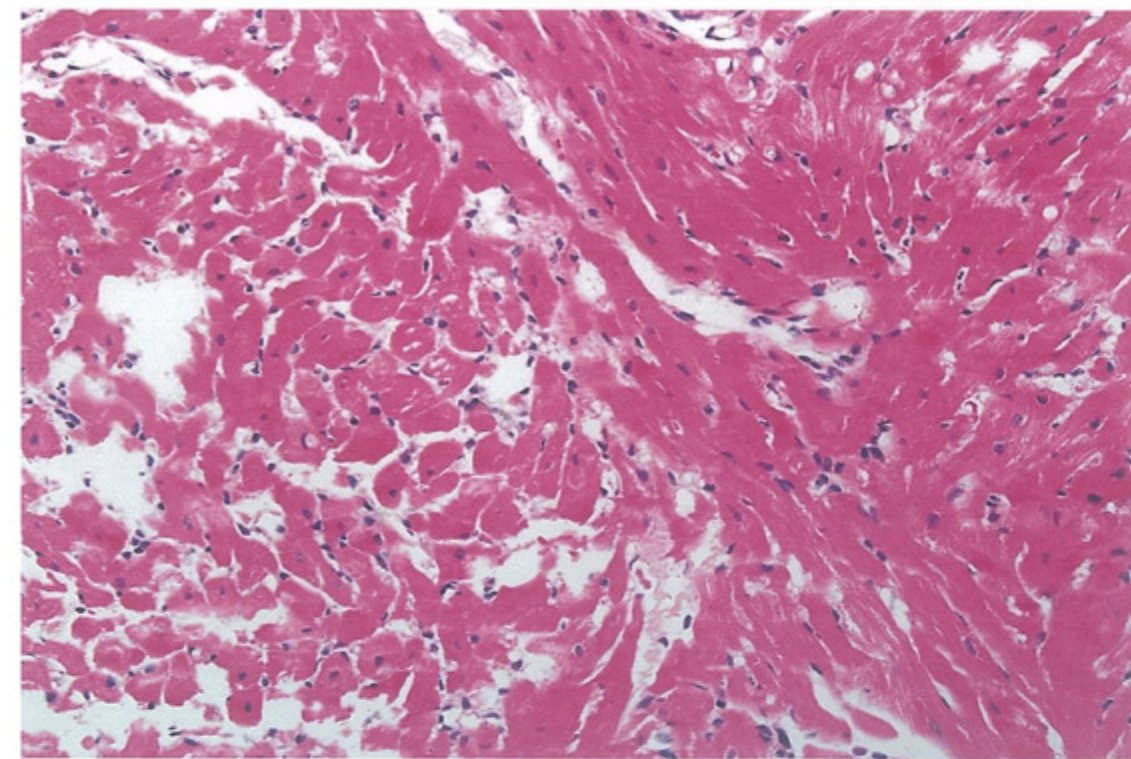

**TAX 5  $\mu$ M**

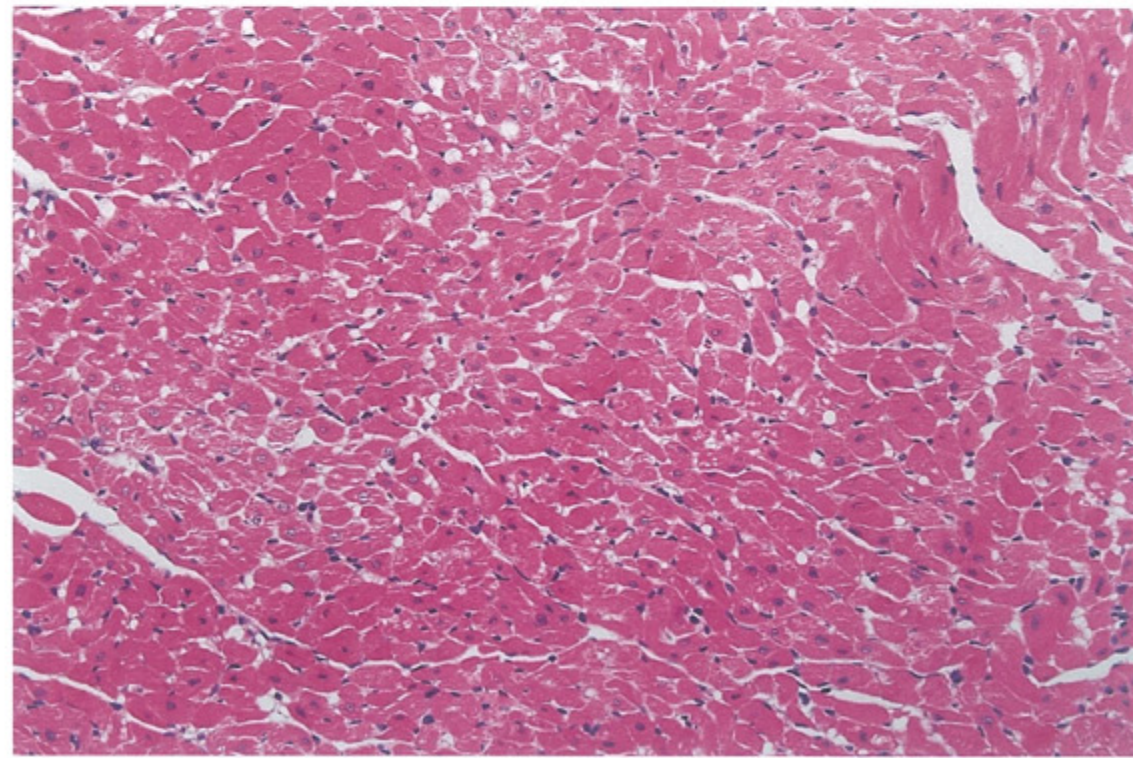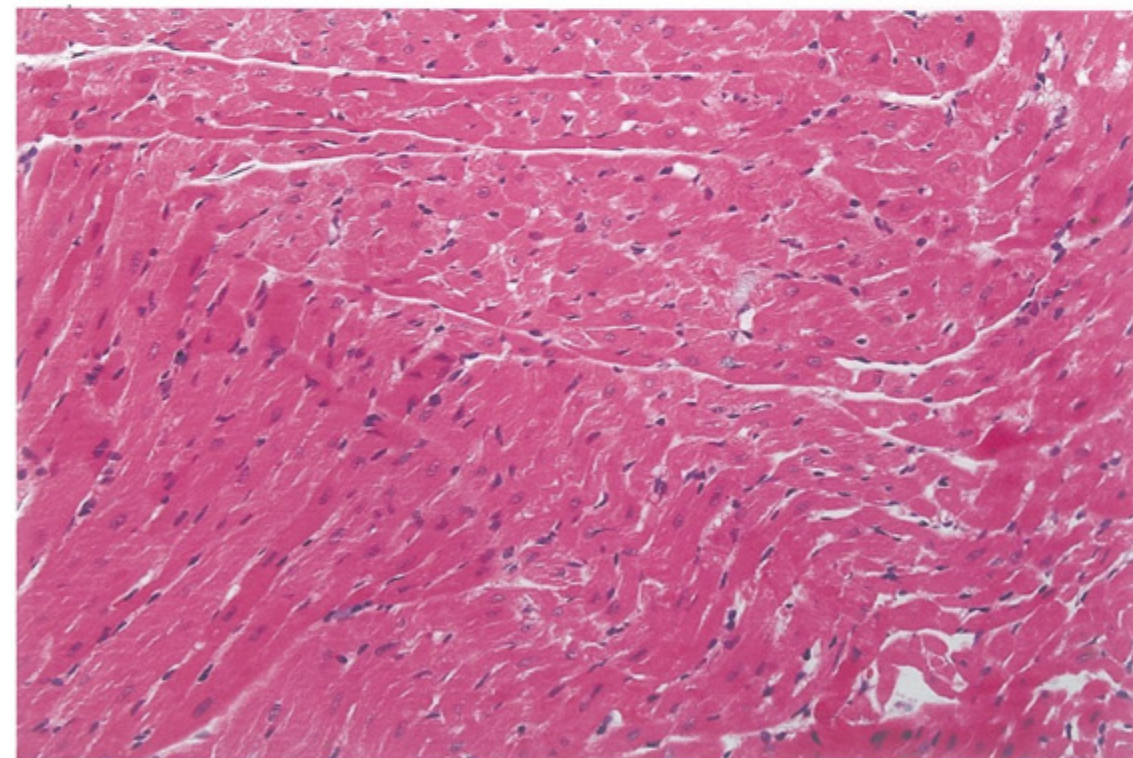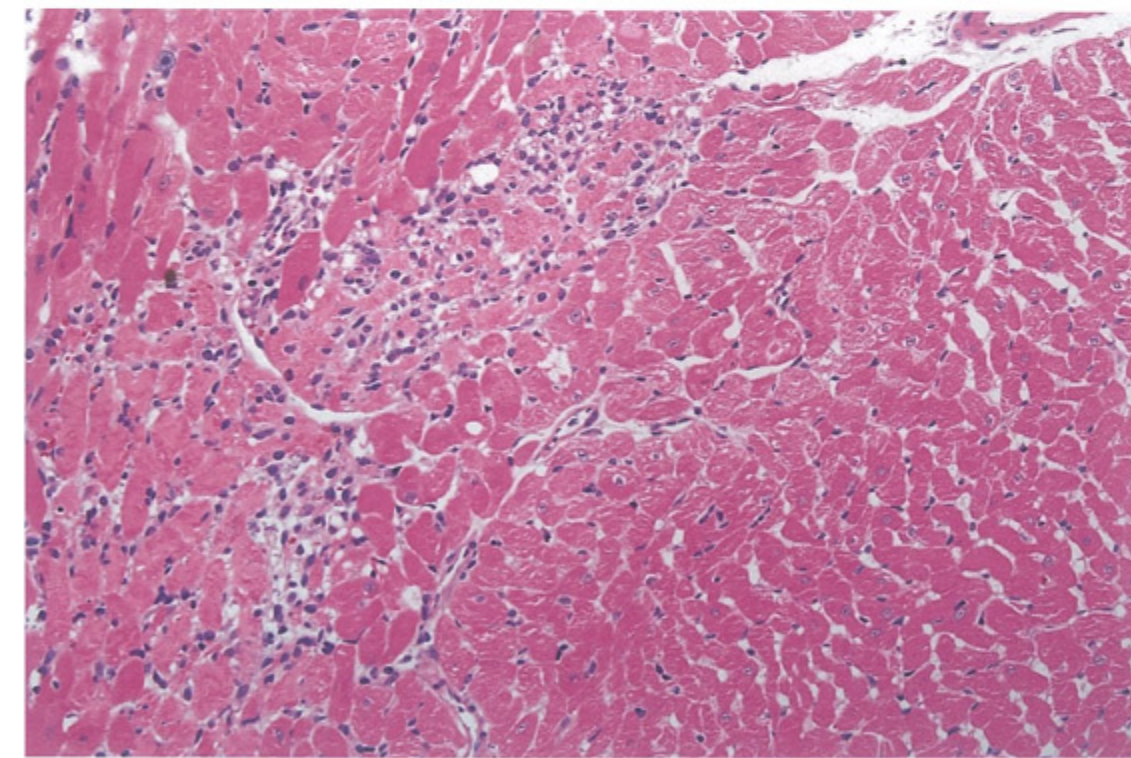

**TAX 15  $\mu$ M**

Supplement: Supplemental Information 4 [file peerj-07-6383-s004.pdf]
