# Supplementary material for: Taxifolin protects rat against myocardial ischemia/reperfusion injury by modulating the mitochondrial apoptosis pathway
Source: PeerJ. 2019 Jan 31;7:e6383. doi: 10.7717/peerj.6383 (PMC6360081; doi:10.7717/peerj.6383)
Supplement: Supplemental Information 5 [file peerj-07-6383-s005.pdf]

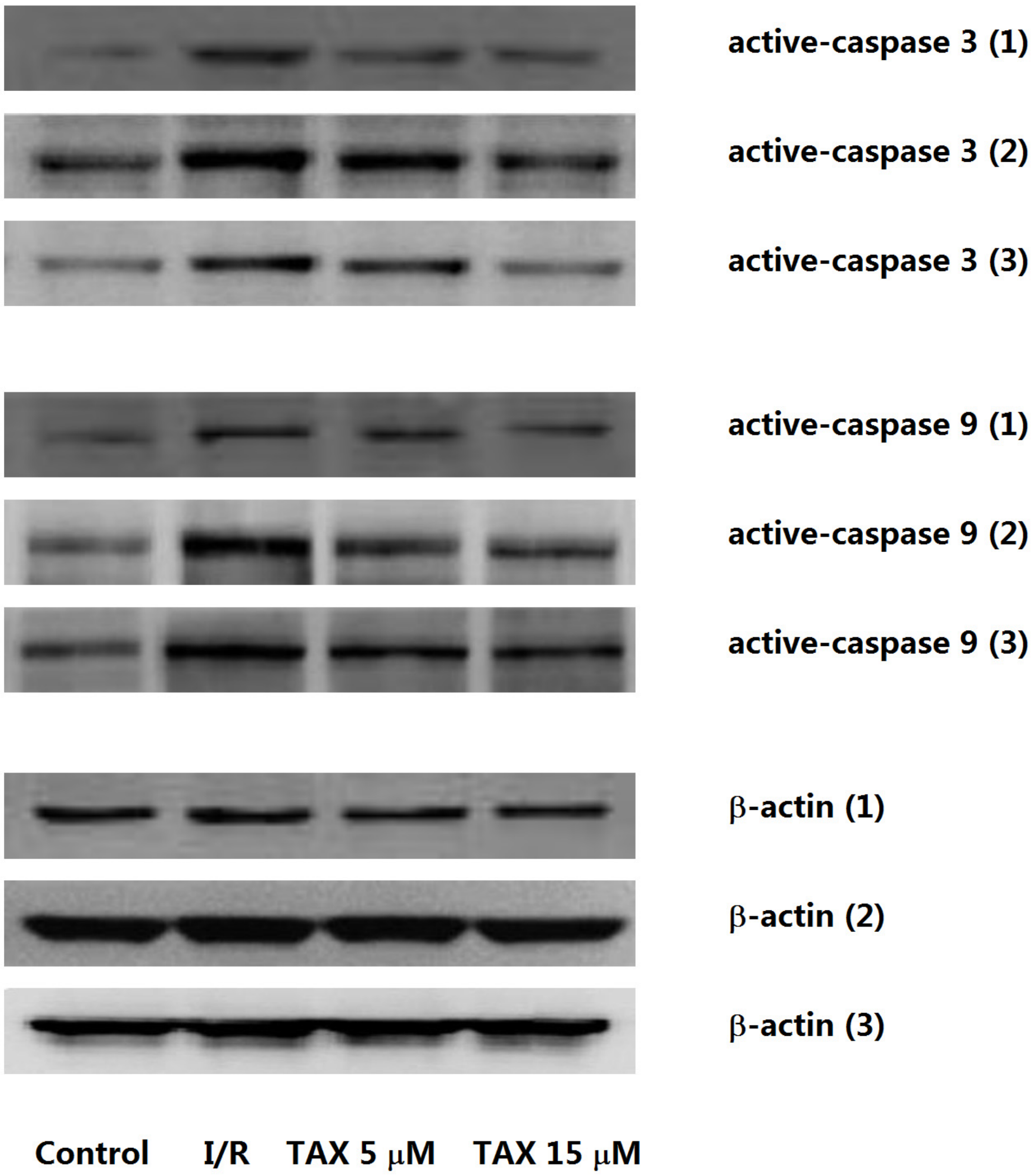
active-caspase 3 (1)
active-caspase 3 (2)
active-caspase 3 (3)
active-caspase 9 (1)
active-caspase 9 (2)
active-caspase 9 (3)
β-actin (1)
β-actin (2)
β-actin (3)
Control
I/R
TAX 5 μM
TAX 15 μM
