# Supplementary material for: Taxifolin protects rat against myocardial ischemia/reperfusion injury by modulating the mitochondrial apoptosis pathway
Source: PeerJ. 2019 Jan 31;7:e6383. doi: 10.7717/peerj.6383 (PMC6360081; doi:10.7717/peerj.6383)
Supplement: Supplemental Information 6 [file peerj-07-6383-s006.zip › Statistical Reporting/Analysis results/Word file form/BAX.doc]

ONEWAY Bax BY Group
  /STATISTICS HOMOGENEITY
  /MISSING ANALYSIS
  /POSTHOC=LSD ALPHA(0.05).

Oneway

C:\Users\Administrator\Desktop\Statistical Reporting\Bax.sav

Test of Homogeneity of Variances	
Bax  	
Levene Statistic	df1	df2	Sig.	
.360	3	20	.782	

ANOVA	
Bax  	
	Sun of Squares	df	Mean Square	F	Sig.	
Between Groups	3528333.614	3	1176111.205	11.495	.000	
Within Groups	2046217.688	20	102310.884			
Total	5574551.301	23				

Post Hoc Tests
Multiple Comparisons	
Dependent Variable: Bax	
LSD  	
(I) Group	(J) Group	Mean Difference (I-J)	Std. Error	Sig.	95% Confidence interval	
					Lower bound	Upper Bound	
1	2	-1011.79639*	177.95408	.000	-1383.0021	-640.5907	
	3	-618.12941*	193.68529	.005	-1022.1498	-214.1090	
	4	-353.58113	184.67168	.070	-738.7995	31.6372	
2	1	1011.79639*	177.95408	.000	640.5907	1383.0021	
	3	393.66698*	187.29126	.048	2.9842	784.3497	
	4	658.21526*	177.95408	.001	287.0096	1029.4210	
3	1	618.12941*	193.68529	.005	214.1090	1022.1498	
	2	-393.66698*	187.29126	.048	-784.3497	-2.9842	
	4	264.54828	193.68529	.187	-139.4722	668.5687	
4	1	353.58113	184.67168	.070	-31.6372	738.7995	
	2	-658.21526*	177.95408	.001	-1029.4210	-287.0096	
	3	-264.54828	193.68529	.187	-668.5687	139.4722	

*. The mean difference is significant at the 0.05 level.	
