# Supplementary material for: Taxifolin protects rat against myocardial ischemia/reperfusion injury by modulating the mitochondrial apoptosis pathway
Source: PeerJ. 2019 Jan 31;7:e6383. doi: 10.7717/peerj.6383 (PMC6360081; doi:10.7717/peerj.6383)
Supplement: Supplemental Information 6 [file peerj-07-6383-s006.zip › Statistical Reporting/Analysis results/Word file form/Bcl2.doc]

ONEWAY Bcl2 BY Group
  /STATISTICS HOMOGENEITY
  /MISSING ANALYSIS
  /POSTHOC=LSD ALPHA(0.05).

Oneway

C:\Users\Administrator\Desktop\Statistical Reporting\Bcl-2.sav

Test of Homogeneity of Variances	
Bcl2  	
Levene Statistic	df1	df2	Sig.	
2.130	3	20	.128	

ANOVA	
Bcl2  	
	Sun of Squares	df	Mean Square	F	Sig.	
Between Groups	10222417.657	3	3407472.552	11.935	.000	
Within Groups	5709879.825	20	285493.991			
Total	15932297.482	23				

Post Hoc Tests
Multiple Comparisons	
Dependent Variable: Bcl2  	
LSD  	
(I) Group	(J) Group	Mean Difference (I-J)	Std. Error	Sig.	95% Confidence interval	
					Lower Bound	Upper Bound	
1	2	1561.37881*	297.26619	.000	941.2924	2181.4652	
	3	1224.49167*	323.54463	.001	549.5894	1899.3939	
	4	312.37167	308.48770	.323	-331.1224	955.8657	
2	1	-1561.37881*	297.26619	.000	-2181.4652	-941.2924	
	3	-336.88714	312.86364	.294	-989.5092	315.7350	
	4	-1249.00714*	297.26619	.000	-1869.0935	-628.9207	
3	1	-1224.49167*	323.54463	.001	-1899.3939	-549.5894	
	2	336.88714	312.86364	.294	-315.7350	989.5092	
	4	-912.12000*	323.54463	.011	-1587.0223	-237.2177	
4	1	-312.37167	308.48770	.323	-955.8657	331.1224	
	2	1249.00714*	297.26619	.000	628.9207	1869.0935	
	3	912.12000*	323.54463	.011	237.2177	1587.0223	

*. The mean difference is significant at the 0.05 level.	
