# Supplementary material for: Taxifolin protects rat against myocardial ischemia/reperfusion injury by modulating the mitochondrial apoptosis pathway
Source: PeerJ. 2019 Jan 31;7:e6383. doi: 10.7717/peerj.6383 (PMC6360081; doi:10.7717/peerj.6383)
Supplement: Supplemental Information 6 [file peerj-07-6383-s006.zip › Statistical Reporting/Analysis results/Word file form/Caspase 3.doc]

ONEWAY Caspase3 BY Group
  /STATISTICS HOMOGENEITY
  /MISSING ANALYSIS
  /POSTHOC=LSD ALPHA(0.05).

Oneway

C:\Users\Administrator\Desktop\Statistical Reporting\Caspase 3.sav

Test of Homogeneity of Variances	
Caspase3  	
Levene Statistic	df1	df2	Sig.	
3.697	3	8	.062	

ANOVA	
Caspase3  	
	Sun of Squares	df	Mean Square	F	Sig.	
Between Groups	2.010	3	.670	12.548	.002	
Within Groups	.427	8	.053			
Total	2.438	11				

Post Hoc Tests
Multiple Comparisons	
Dependent Variable: Caspase3  	
LSD  	
(I) Group	(J) Group	Mean Difference (I-J)	Std. Error	Sig.	95% Confidence interval	
					Lower Bound	Upper Bound	
1.00	2.00	-1.11333*	.18869	.000	-1.5485	-.6782	
	3.00	-.76000*	.18869	.004	-1.1951	-.3249	
	4.00	-.45000*	.18869	.044	-.8851	-.0149	
2.00	1.00	1.11333*	.18869	.000	.6782	1.5485	
	3.00	.35333	.18869	.048	-.0818	.7885	
	4.00	.66333*	.18869	.008	.2282	1.0985	
3.00	1.00	.76000*	.18869	.004	.3249	1.1951	
	2.00	-.35333	.18869	.098	-.7885	.0818	
	4.00	.31000	.18869	.139	-.1251	.7451	
4.00	1.00	.45000*	.18869	.044	.0149	.8851	
	2.00	-.66333*	.18869	.008	-1.0985	-.2282	
	3.00	-.31000	.18869	.139	-.7451	.1251	

*. The mean difference is significant at the 0.05 level.	
