# Supplementary material for: Taxifolin protects rat against myocardial ischemia/reperfusion injury by modulating the mitochondrial apoptosis pathway
Source: PeerJ. 2019 Jan 31;7:e6383. doi: 10.7717/peerj.6383 (PMC6360081; doi:10.7717/peerj.6383)
Supplement: Supplemental Information 6 [file peerj-07-6383-s006.zip › Statistical Reporting/Analysis results/Word file form/Caspase 9.doc]

ONEWAY Caspase9 BY Group
  /STATISTICS HOMOGENEITY
  /MISSING ANALYSIS
  /POSTHOC=LSD ALPHA(0.05).
Oneway

C:\Users\Administrator\Desktop\Statistical Reporting\Caspase 9.sav

Test of Homogeneity of Variances	
Caspase9  	
Levene Statistic	df1	df2	Sig.	
3.405	3	8	.074	

ANOVA	
Caspase9  	
	Sun of Squares	df	Mean Square	F	Sig.	
Between Groups	.717	3	.239	12.543	.002	
Within Groups	.153	8	.019			
Total	.870	11				

Post Hoc Tests
Multiple Comparisons	
Dependent Variable: Caspase9	
LSD  	
(I) Group	(J) Group	Mean Difference (I-J)	Std. Error	Sig.	95% Confidence interval	
					Lower Bound	Lower Bound	
1.00	2.00	-.64000*	.11274	.000	-.9000	-.3800	
	3.00	-.42667*	.11274	.005	-.6867	-.1667	
	4.00	-.16667	.11274	.178	-.4267	.0933	
2.00	1.00	.64000*	.11274	.000	.3800	.9000	
	3.00	.21333	.11274	.095	-.0467	.4733	
	4.00	.47333*	.11274	.003	.2133	.7333	
3.00	1.00	.42667*	.11274	.005	.1667	.6867	
	2.00	-.21333	.11274	.095	-.4733	.0467	
	4.00	.26000*	.11274	.050	.0000	.5200	
4.00	1.00	.16667	.11274	.178	-.0933	.4267	
	2.00	-.47333*	.11274	.003	-.7333	-.2133	
	3.00	-.26000*	.11274	.050	-.5200	.0000	

*. The mean difference is significant at the 0.05 level.	
