# Supplementary material for: Taxifolin protects rat against myocardial ischemia/reperfusion injury by modulating the mitochondrial apoptosis pathway
Source: PeerJ. 2019 Jan 31;7:e6383. doi: 10.7717/peerj.6383 (PMC6360081; doi:10.7717/peerj.6383)
Supplement: Supplemental Information 6 [file peerj-07-6383-s006.zip › Statistical Reporting/Analysis results/Word file form/CK-MB.doc]

ONEWAY Time25min Time63min Time90min Time120min BY Group
  /STATISTICS HOMOGENEITY
  /MISSING ANALYSIS
  /POSTHOC=LSD ALPHA(0.05).

Oneway

C:\Users\Administrator\Desktop\Statistical Reporting\CK-MB.sav

Test of Homogeneity of Variances	
	Levene Statistic	df1	df2	Sig.	
Time25min	1.063	3	22	.385	
Time63min	.208	3	22	.890	
Time90min	.089	3	15	.965	
Time120min	.072	3	21	.975	

ANOVA	
	Sun of Squares	df	Mean Square	F	Sig.	
Time25min	Between Groups	36979.864	3	12326.621	4.405	.014	
	Within Groups	61567.099	22	2798.504			
	Total	98546.963	25				
Time63min	Between Groups	13818.057	3	4606.019	1.676	.201	
	Within Groups	60453.945	22	2747.907			
	Total	74272.001	25				
Time90min	Between Groups	124666.939	3	41555.646	7.346	.003	
	Within Groups	84853.405	15	5656.894			
	Total	209520.344	18				
Time120min	Between Groups	95509.684	3	31836.561	6.996	.002	
	Within Groups	95559.741	21	4550.464			
	Total	191069.426	24				

Post Hoc Tests
Multiple Comparisons	
LSD  	
Dependent Variable	(I) Group	(J) Group	Mean Difference (I-J)	Std. Error	Sig.	95% Confidence interval	
						Lower bound	Lower bound	
Time25min	1.00	2.00	-98.25452*	29.43134	.003	-159.2914	-37.2177	
		3.00	-37.24500	30.54234	.236	-100.5859	26.0959	
		4.00	-17.38167	29.43134	.561	-78.4185	43.6552	
	2.00	1.00	98.25452*	29.43134	.003	37.2177	159.2914	
		3.00	61.00952	29.43134	.050	-.0273	122.0464	
		4.00	80.87286*	28.27672	.009	22.2305	139.5152	
	3.00	1.00	37.24500	30.54234	.236	-26.0959	100.5859	
		2.00	-61.00952	29.43134	.050	-122.0464	.0273	
		4.00	19.86333	29.43134	.507	-41.1735	80.9002	
	4.00	1.00	17.38167	29.43134	.561	-43.6552	78.4185	
		2.00	-80.87286*	28.27672	.009	-139.5152	-22.2305	
		3.00	-19.86333	29.43134	.507	-80.9002	41.1735	
Time63min	1.00	2.00	-35.64898	29.16406	.235	-96.1315	24.8336	
		3.00	-54.62667	30.26498	.085	-117.3924	8.1391	
		4.00	-.53198	29.16406	.986	-61.0145	59.9506	
	2.00	1.00	35.64898	29.16406	.235	-24.8336	96.1315	
		3.00	-18.97769	29.16406	.522	-79.4603	41.5049	
		4.00	35.11700	28.01992	.223	-22.9928	93.2268	
	3.00	1.00	54.62667	30.26498	.085	-8.1391	117.3924	
		2.00	18.97769	29.16406	.522	-41.5049	79.4603	
		4.00	54.09469	29.16406	.077	-6.3879	114.5773	
	4.00	1.00	.53198	29.16406	.986	-59.9506	61.0145	
		2.00	-35.11700	28.01992	.223	-93.2268	22.9928	
		3.00	-54.09469	29.16406	.077	-114.5773	6.3879	
Time90min	1.00	2.00	-217.26750*	48.54935	.000	-320.7480	-113.7870	
		3.00	-78.21500	53.18314	.162	-191.5722	35.1422	
		4.00	-84.17850	50.45396	.116	-191.7186	23.3616	
	2.00	1.00	217.26750*	48.54935	.000	113.7870	320.7480	
		3.00	139.05250*	48.54935	.012	35.5720	242.5330	
		4.00	133.08900*	45.54332	.011	36.0157	230.1623	
	3.00	1.00	78.21500	53.18314	.162	-35.1422	191.5722	
		2.00	-139.05250*	48.54935	.012	-242.5330	-35.5720	
		4.00	-5.96350	50.45396	.907	-113.5036	101.5766	
	4.00	1.00	84.17850	50.45396	.116	-23.3616	191.7186	
		2.00	-133.08900*	45.54332	.011	-230.1623	-36.0157	
		3.00	5.96350	50.45396	.907	-101.5766	113.5036	
Time120min	1.00	2.00	-155.90000*	37.52968	.000	-233.9472	-77.8528	
		3.00	-60.34000	40.84732	.154	-145.2866	24.6066	
		4.00	-22.88143	37.52968	.549	-100.9287	55.1658	
	2.00	1.00	155.90000*	37.52968	.000	77.8528	233.9472	
		3.00	95.56000*	39.49885	.025	13.4176	177.7024	
		4.00	133.01857*	36.05735	.001	58.0332	208.0039	
	3.00	1.00	60.34000	40.84732	.154	-24.6066	145.2866	
		2.00	-95.56000*	39.49885	.025	-177.7024	-13.4176	
		4.00	37.45857	39.49885	.354	-44.6838	119.6009	
	4.00	1.00	22.88143	37.52968	.549	-55.1658	100.9287	
		2.00	-133.01857*	36.05735	.001	-208.0039	-58.0332	
		3.00	-37.45857	39.49885	.354	-119.6009	44.6838	

*. The mean difference is significant at the 0.05 level.	
