# Supplementary material for: Taxifolin protects rat against myocardial ischemia/reperfusion injury by modulating the mitochondrial apoptosis pathway
Source: PeerJ. 2019 Jan 31;7:e6383. doi: 10.7717/peerj.6383 (PMC6360081; doi:10.7717/peerj.6383)
Supplement: Supplemental Information 6 [file peerj-07-6383-s006.zip › Statistical Reporting/Analysis results/Word file form/Cyt-c.doc]

ONEWAY Cytc BY Group
  /STATISTICS HOMOGENEITY
  /MISSING ANALYSIS
  /POSTHOC=LSD ALPHA(0.05).

Oneway

C:\Users\Administrator\Desktop\Statistical Reporting\Cyt-c.sav

Test of Homogeneity of Variances	
Cytc  	
Levene Statistic	df1	df2	Sig.	
4.053	3	20	.021	

ANOVA	
Cytc  	
	Sun of Squares	df	Mean Square	F	Sig.	
Between Groups	541.734	3	180.578	14.727	.000	
Within Groups	245.234	20	12.262			
Total	786.969	23				

Post Hoc Tests
Multiple Comparisons	
Dependent Variable: Cytc  	
LSD  	
(I) Group	(J) Group	Mean Difference (I-J)	Std. Error	Sig.	95% Confidence interval	
					Lower Bound	Lower Bound	
1	2	-12.37596*	1.94815	.000	-16.4397	-8.3122	
	3	-5.24054*	2.12037	.023	-9.6635	-.8175	
	4	-3.30289	2.02169	.118	-7.5201	.9143	
2	1	12.37596*	1.94815	.000	8.3122	16.4397	
	3	7.13542*	2.05037	.002	2.8584	11.4124	
	4	9.07307*	1.94815	.000	5.0093	13.1368	
3	1	5.24054*	2.12037	.023	.8175	9.6635	
	2	-7.13542*	2.05037	.002	-11.4124	-2.8584	
	4	1.93764	2.12037	.372	-2.4854	6.3607	
4	1	3.30289	2.02169	.118	-.9143	7.5201	
	2	-9.07307*	1.94815	.000	-13.1368	-5.0093	
	3	-1.93764	2.12037	.372	-6.3607	2.4854	

*. The mean difference is significant at the 0.05 level.	
