# Supplementary material for: Taxifolin protects rat against myocardial ischemia/reperfusion injury by modulating the mitochondrial apoptosis pathway
Source: PeerJ. 2019 Jan 31;7:e6383. doi: 10.7717/peerj.6383 (PMC6360081; doi:10.7717/peerj.6383)
Supplement: Supplemental Information 6 [file peerj-07-6383-s006.zip › Statistical Reporting/Analysis results/Word file form/dpdtmax.doc]

ONEWAY Time10min Time20min Time30min Time60min Time70min Time80min Time90min Time100min Time110min Time120min BY Group
  /STATISTICS HOMOGENEITY
  /MISSING ANALYSIS
  /POSTHOC=LSD ALPHA(0.05).

Oneway

C:\Users\Administrator\Desktop\Statistical Reporting\dpdt max .sav

¤è®t齐©Ê检验	
	Levene 统计¶q	df1	df2	显µÛ©Ê	
Time10min	1.999	3	19	.148	
Time20min	3.612	3	19	.032	
Time30min	2.221	3	19	.119	
Time60min	.183	3	19	.907	
Time70min	12.230	3	19	.000	
Time80min	1.897	3	19	.164	
Time90min	1.073	3	19	.384	
Time100min	.785	3	19	.517	
Time110min	.005	3	19	.999	
Time120min	1.956	3	19	.155	

单¦]¯À¤è®t¤ÀªR	
	¥­¤è©M	df	§¡¤è	F	显µÛ©Ê	
Time10min	组间	343036.959	3	114345.653	1.364	.284	
	组内	1592428.867	19	83812.046			
	总数	1935465.826	22				
Time20min	组间	417175.772	3	139058.591	1.117	.367	
	组内	2365033.967	19	124475.472			
	总数	2782209.739	22				
Time30min	组间	397587.323	3	132529.108	1.344	.290	
	组内	1873959.633	19	98629.454			
	总数	2271546.957	22				
Time60min	组间	585563.642	3	195187.881	1.486	.250	
	组内	2495962.967	19	131366.472			
	总数	3081526.609	22				
Time70min	组间	384007.301	3	128002.434	1.883	.167	
	组内	1291305.133	19	67963.428			
	总数	1675312.435	22				
Time80min	组间	872712.452	3	290904.151	7.844	.001	
	组内	704671.200	19	37087.958			
	总数	1577383.652	22				
Time90min	组间	422243.706	3	140747.902	3.504	.036	
	组内	763292.033	19	40173.265			
	总数	1185535.739	22				
Time100min	组间	750132.546	3	250044.182	5.760	.006	
	组内	824815.367	19	43411.335			
	总数	1574947.913	22				
Time110min	组间	796542.112	3	265514.037	12.892	.000	
	组内	391297.367	19	20594.598			
	总数	1187839.478	22				
Time120min	组间	1758531.670	3	586177.223	33.966	.000	
	组内	327897.200	19	17257.747			
	总数	2086428.870	22				

Post Hoc Tests
¦h­«¤ñ较	
LSD  	
¦]变¶q	(I) Group	(J) Group	§¡­È®t (I-J)	标­ã误	显µÛ©Ê	95% ¸m«H区间	
						¤U­­	¤W­­	
Time10min	1.00	2.00	-208.16667	167.14469	.228	-558.0045	141.6712	
		3.00	141.60000	175.30283	.429	-225.3130	508.5130	
		4.00	-32.16667	167.14469	.849	-382.0045	317.6712	
	2.00	1.00	208.16667	167.14469	.228	-141.6712	558.0045	
		3.00	349.76667	175.30283	.061	-17.1464	716.6797	
		4.00	176.00000	167.14469	.306	-173.8379	525.8379	
	3.00	1.00	-141.60000	175.30283	.429	-508.5130	225.3130	
		2.00	-349.76667	175.30283	.061	-716.6797	17.1464	
		4.00	-173.76667	175.30283	.334	-540.6797	193.1464	
	4.00	1.00	32.16667	167.14469	.849	-317.6712	382.0045	
		2.00	-176.00000	167.14469	.306	-525.8379	173.8379	
		3.00	173.76667	175.30283	.334	-193.1464	540.6797	
Time20min	1.00	2.00	-295.33333	203.69542	.163	-721.6727	131.0061	
		3.00	37.36667	213.63756	.863	-409.7819	484.5152	
		4.00	-181.66667	203.69542	.384	-608.0061	244.6727	
	2.00	1.00	295.33333	203.69542	.163	-131.0061	721.6727	
		3.00	332.70000	213.63756	.136	-114.4485	779.8485	
		4.00	113.66667	203.69542	.583	-312.6727	540.0061	
	3.00	1.00	-37.36667	213.63756	.863	-484.5152	409.7819	
		2.00	-332.70000	213.63756	.136	-779.8485	114.4485	
		4.00	-219.03333	213.63756	.318	-666.1819	228.1152	
	4.00	1.00	181.66667	203.69542	.384	-244.6727	608.0061	
		2.00	-113.66667	203.69542	.583	-540.0061	312.6727	
		3.00	219.03333	213.63756	.318	-228.1152	666.1819	
Time30min	1.00	2.00	-276.66667	181.31874	.144	-656.1711	102.8378	
		3.00	75.80000	190.16870	.695	-322.2277	473.8277	
		4.00	-112.50000	181.31874	.542	-492.0045	267.0045	
	2.00	1.00	276.66667	181.31874	.144	-102.8378	656.1711	
		3.00	352.46667	190.16870	.079	-45.5610	750.4943	
		4.00	164.16667	181.31874	.377	-215.3378	543.6711	
	3.00	1.00	-75.80000	190.16870	.695	-473.8277	322.2277	
		2.00	-352.46667	190.16870	.079	-750.4943	45.5610	
		4.00	-188.30000	190.16870	.335	-586.3277	209.7277	
	4.00	1.00	112.50000	181.31874	.542	-267.0045	492.0045	
		2.00	-164.16667	181.31874	.377	-543.6711	215.3378	
		3.00	188.30000	190.16870	.335	-209.7277	586.3277	
Time60min	1.00	2.00	244.16667	209.25779	.258	-193.8149	682.1483	
		3.00	353.86667	219.47142	.123	-105.4923	813.2256	
		4.00	-31.00000	209.25779	.884	-468.9816	406.9816	
	2.00	1.00	-244.16667	209.25779	.258	-682.1483	193.8149	
		3.00	109.70000	219.47142	.623	-349.6590	569.0590	
		4.00	-275.16667	209.25779	.204	-713.1483	162.8149	
	3.00	1.00	-353.86667	219.47142	.123	-813.2256	105.4923	
		2.00	-109.70000	219.47142	.623	-569.0590	349.6590	
		4.00	-384.86667	219.47142	.096	-844.2256	74.4923	
	4.00	1.00	31.00000	209.25779	.884	-406.9816	468.9816	
		2.00	275.16667	209.25779	.204	-162.8149	713.1483	
		3.00	384.86667	219.47142	.096	-74.4923	844.2256	
Time70min	1.00	2.00	334.66667*	150.51404	.039	19.6372	649.6962	
		3.00	262.70000	157.86046	.112	-67.7057	593.1057	
		4.00	135.50000	150.51404	.379	-179.5295	450.5295	
	2.00	1.00	-334.66667*	150.51404	.039	-649.6962	-19.6372	
		3.00	-71.96667	157.86046	.654	-402.3724	258.4391	
		4.00	-199.16667	150.51404	.201	-514.1962	115.8628	
	3.00	1.00	-262.70000	157.86046	.112	-593.1057	67.7057	
		2.00	71.96667	157.86046	.654	-258.4391	402.3724	
		4.00	-127.20000	157.86046	.430	-457.6057	203.2057	
	4.00	1.00	-135.50000	150.51404	.379	-450.5295	179.5295	
		2.00	199.16667	150.51404	.201	-115.8628	514.1962	
		3.00	127.20000	157.86046	.430	-203.2057	457.6057	
Time80min	1.00	2.00	536.00000*	111.18747	.000	303.2820	768.7180	
		3.00	283.26667*	116.61440	.025	39.1899	527.3434	
		4.00	225.00000	111.18747	.057	-7.7180	457.7180	
	2.00	1.00	-536.00000*	111.18747	.000	-768.7180	-303.2820	
		3.00	-252.73333*	116.61440	.043	-496.8101	-8.6566	
		4.00	-311.00000*	111.18747	.011	-543.7180	-78.2820	
	3.00	1.00	-283.26667*	116.61440	.025	-527.3434	-39.1899	
		2.00	252.73333*	116.61440	.043	8.6566	496.8101	
		4.00	-58.26667	116.61440	.623	-302.3434	185.8101	
	4.00	1.00	-225.00000	111.18747	.057	-457.7180	7.7180	
		2.00	311.00000*	111.18747	.011	78.2820	543.7180	
		3.00	58.26667	116.61440	.623	-185.8101	302.3434	
Time90min	1.00	2.00	292.66667*	115.71987	.020	50.4622	534.8711	
		3.00	272.40000*	121.36802	.037	18.3738	526.4262	
		4.00	26.50000	115.71987	.821	-215.7045	268.7045	
	2.00	1.00	-292.66667*	115.71987	.020	-534.8711	-50.4622	
		3.00	-20.26667	121.36802	.869	-274.2929	233.7595	
		4.00	-266.16667*	115.71987	.033	-508.3711	-23.9622	
	3.00	1.00	-272.40000*	121.36802	.037	-526.4262	-18.3738	
		2.00	20.26667	121.36802	.869	-233.7595	274.2929	
		4.00	-245.90000	121.36802	.057	-499.9262	8.1262	
	4.00	1.00	-26.50000	115.71987	.821	-268.7045	215.7045	
		2.00	266.16667*	115.71987	.033	23.9622	508.3711	
		3.00	245.90000	121.36802	.057	-8.1262	499.9262	
Time100min	1.00	2.00	492.16667*	120.29316	.001	240.3902	743.9432	
		3.00	282.40000*	126.16453	.037	18.3346	546.4654	
		4.00	191.66667	120.29316	.128	-60.1098	443.4432	
	2.00	1.00	-492.16667*	120.29316	.001	-743.9432	-240.3902	
		3.00	-209.76667	126.16453	.113	-473.8321	54.2987	
		4.00	-300.50000*	120.29316	.022	-552.2765	-48.7235	
	3.00	1.00	-282.40000*	126.16453	.037	-546.4654	-18.3346	
		2.00	209.76667	126.16453	.113	-54.2987	473.8321	
		4.00	-90.73333	126.16453	.481	-354.7987	173.3321	
	4.00	1.00	-191.66667	120.29316	.128	-443.4432	60.1098	
		2.00	300.50000*	120.29316	.022	48.7235	552.2765	
		3.00	90.73333	126.16453	.481	-173.3321	354.7987	
Time110min	1.00	2.00	485.00000*	82.85449	.000	311.5836	658.4164	
		3.00	398.73333*	86.89852	.000	216.8526	580.6140	
		4.00	255.83333*	82.85449	.006	82.4169	429.2498	
	2.00	1.00	-485.00000*	82.85449	.000	-658.4164	-311.5836	
		3.00	-86.26667	86.89852	.333	-268.1474	95.6140	
		4.00	-229.16667*	82.85449	.012	-402.5831	-55.7502	
	3.00	1.00	-398.73333*	86.89852	.000	-580.6140	-216.8526	
		2.00	86.26667	86.89852	.333	-95.6140	268.1474	
		4.00	-142.90000	86.89852	.117	-324.7807	38.9807	
	4.00	1.00	-255.83333*	82.85449	.006	-429.2498	-82.4169	
		2.00	229.16667*	82.85449	.012	55.7502	402.5831	
		3.00	142.90000	86.89852	.117	-38.9807	324.7807	
Time120min	1.00	2.00	717.66667*	75.84578	.000	558.9196	876.4137	
		3.00	549.93333*	79.54773	.000	383.4380	716.4286	
		4.00	277.00000*	75.84578	.002	118.2530	435.7470	
	2.00	1.00	-717.66667*	75.84578	.000	-876.4137	-558.9196	
		3.00	-167.73333*	79.54773	.048	-334.2286	-1.2380	
		4.00	-440.66667*	75.84578	.000	-599.4137	-281.9196	
	3.00	1.00	-549.93333*	79.54773	.000	-716.4286	-383.4380	
		2.00	167.73333*	79.54773	.048	1.2380	334.2286	
		4.00	-272.93333*	79.54773	.003	-439.4286	-106.4380	
	4.00	1.00	-277.00000*	75.84578	.002	-435.7470	-118.2530	
		2.00	440.66667*	75.84578	.000	281.9196	599.4137	
		3.00	272.93333*	79.54773	.003	106.4380	439.4286	

*. §¡­È®tªº显µÛ©Ê¤ô¥­为 0.05¡C	
