# Supplementary material for: Taxifolin protects rat against myocardial ischemia/reperfusion injury by modulating the mitochondrial apoptosis pathway
Source: PeerJ. 2019 Jan 31;7:e6383. doi: 10.7717/peerj.6383 (PMC6360081; doi:10.7717/peerj.6383)
Supplement: Supplemental Information 6 [file peerj-07-6383-s006.zip › Statistical Reporting/Analysis results/Word file form/dpdtmin.doc]

ONEWAY Time10min Time20min Time30min Time60min Time70min Time80min Time90min Time100min Time110min Time120min BY Group
  /STATISTICS HOMOGENEITY
  /MISSING ANALYSIS
  /POSTHOC=LSD ALPHA(0.05).

Oneway

C:\Users\Administrator\Desktop\Statistical Reporting\dpdt min .sav

¤è®t齐©Ê检验	
	Levene 统计¶q	df1	df2	显µÛ©Ê	
Time10min	3.004	3	19	.056	
Time20min	.407	3	19	.749	
Time30min	3.580	3	19	.033	
Time60min	1.393	3	19	.275	
Time70min	4.815	3	19	.012	
Time80min	1.860	3	19	.171	
Time90min	1.516	3	19	.243	
Time100min	1.346	3	19	.289	
Time110min	2.451	3	19	.095	
Time120min	5.005	3	19	.010	

单¦]¯À¤è®t¤ÀªR	
	¥­¤è©M	df	§¡¤è	F	显µÛ©Ê	
Time10min	组间	44560.090	3	14853.363	.211	.888	
	组内	1337608.867	19	70400.467			
	总数	1382168.957	22				
Time20min	组间	226846.439	3	75615.480	.740	.541	
	组内	1941423.300	19	102180.174			
	总数	2168269.739	22				
Time30min	组间	94756.990	3	31585.663	.206	.891	
	组内	2912775.967	19	153303.998			
	总数	3007532.957	22				
Time60min	组间	422762.959	3	140920.986	1.792	.183	
	组内	1494410.867	19	78653.204			
	总数	1917173.826	22				
Time70min	组间	256078.251	3	85359.417	2.326	.107	
	组内	697194.967	19	36694.472			
	总数	953273.217	22				
Time80min	组间	385228.584	3	128409.528	1.628	.216	
	组内	1498424.633	19	78864.454			
	总数	1883653.217	22				
Time90min	组间	89488.764	3	29829.588	.750	.536	
	组内	756175.500	19	39798.711			
	总数	845664.264	22				
Time100min	组间	361915.882	3	120638.627	3.188	.047	
	组内	718888.167	19	37836.219			
	总数	1080804.049	22				
Time110min	组间	235296.011	3	78432.004	3.005	.056	
	组内	495852.167	19	26097.482			
	总数	731148.177	22				
Time120min	组间	526506.940	3	175502.313	9.138	.001	
	组内	364912.333	19	19205.912			
	总数	891419.273	22				

Post Hoc Tests
¦h­«¤ñ较	
LSD  	
¦]变¶q	(I) Group	(J) Group	§¡­È®t (I-J)	标­ã误	显µÛ©Ê	95% ¸m«H区间	
						¤U­­	¤W­­	
Time10min	1.00	2.00	-40.83333	153.18884	.793	-361.4613	279.7946	
		3.00	-111.23333	160.66582	.497	-447.5108	225.0441	
		4.00	-95.66667	153.18884	.540	-416.2946	224.9613	
	2.00	1.00	40.83333	153.18884	.793	-279.7946	361.4613	
		3.00	-70.40000	160.66582	.666	-406.6774	265.8774	
		4.00	-54.83333	153.18884	.724	-375.4613	265.7946	
	3.00	1.00	111.23333	160.66582	.497	-225.0441	447.5108	
		2.00	70.40000	160.66582	.666	-265.8774	406.6774	
		4.00	15.56667	160.66582	.924	-320.7108	351.8441	
	4.00	1.00	95.66667	153.18884	.540	-224.9613	416.2946	
		2.00	54.83333	153.18884	.724	-265.7946	375.4613	
		3.00	-15.56667	160.66582	.924	-351.8441	320.7108	
Time20min	1.00	2.00	-264.00000	184.55367	.169	-650.2753	122.2753	
		3.00	-199.36667	193.56152	.316	-604.4956	205.7623	
		4.00	-132.00000	184.55367	.483	-518.2753	254.2753	
	2.00	1.00	264.00000	184.55367	.169	-122.2753	650.2753	
		3.00	64.63333	193.56152	.742	-340.4956	469.7623	
		4.00	132.00000	184.55367	.483	-254.2753	518.2753	
	3.00	1.00	199.36667	193.56152	.316	-205.7623	604.4956	
		2.00	-64.63333	193.56152	.742	-469.7623	340.4956	
		4.00	67.36667	193.56152	.732	-337.7623	472.4956	
	4.00	1.00	132.00000	184.55367	.483	-254.2753	518.2753	
		2.00	-132.00000	184.55367	.483	-518.2753	254.2753	
		3.00	-67.36667	193.56152	.732	-472.4956	337.7623	
Time30min	1.00	2.00	-165.00000	226.05604	.474	-638.1407	308.1407	
		3.00	-99.96667	237.08957	.678	-596.2008	396.2675	
		4.00	-139.66667	226.05604	.544	-612.8074	333.4741	
	2.00	1.00	165.00000	226.05604	.474	-308.1407	638.1407	
		3.00	65.03333	237.08957	.787	-431.2008	561.2675	
		4.00	25.33333	226.05604	.912	-447.8074	498.4741	
	3.00	1.00	99.96667	237.08957	.678	-396.2675	596.2008	
		2.00	-65.03333	237.08957	.787	-561.2675	431.2008	
		4.00	-39.70000	237.08957	.869	-535.9342	456.5342	
	4.00	1.00	139.66667	226.05604	.544	-333.4741	612.8074	
		2.00	-25.33333	226.05604	.912	-498.4741	447.8074	
		3.00	39.70000	237.08957	.869	-456.5342	535.9342	
Time60min	1.00	2.00	192.16667	161.91891	.250	-146.7335	531.0668	
		3.00	147.56667	169.82199	.396	-207.8748	503.0082	
		4.00	-150.00000	161.91891	.366	-488.9002	188.9002	
	2.00	1.00	-192.16667	161.91891	.250	-531.0668	146.7335	
		3.00	-44.60000	169.82199	.796	-400.0415	310.8415	
		4.00	-342.16667*	161.91891	.048	-681.0668	-3.2665	
	3.00	1.00	-147.56667	169.82199	.396	-503.0082	207.8748	
		2.00	44.60000	169.82199	.796	-310.8415	400.0415	
		4.00	-297.56667	169.82199	.096	-653.0082	57.8748	
	4.00	1.00	150.00000	161.91891	.366	-188.9002	488.9002	
		2.00	342.16667*	161.91891	.048	3.2665	681.0668	
		3.00	297.56667	169.82199	.096	-57.8748	653.0082	
Time70min	1.00	2.00	276.16667*	110.59607	.022	44.6864	507.6469	
		3.00	188.13333	115.99414	.121	-54.6452	430.9119	
		4.00	88.33333	110.59607	.434	-143.1469	319.8136	
	2.00	1.00	-276.16667*	110.59607	.022	-507.6469	-44.6864	
		3.00	-88.03333	115.99414	.457	-330.8119	154.7452	
		4.00	-187.83333	110.59607	.106	-419.3136	43.6469	
	3.00	1.00	-188.13333	115.99414	.121	-430.9119	54.6452	
		2.00	88.03333	115.99414	.457	-154.7452	330.8119	
		4.00	-99.80000	115.99414	.400	-342.5785	142.9785	
	4.00	1.00	-88.33333	110.59607	.434	-319.8136	143.1469	
		2.00	187.83333	110.59607	.106	-43.6469	419.3136	
		3.00	99.80000	115.99414	.400	-142.9785	342.5785	
Time80min	1.00	2.00	356.66667*	162.13621	.040	17.3117	696.0217	
		3.00	202.36667	170.04989	.249	-153.5519	558.2852	
		4.00	166.66667	162.13621	.317	-172.6883	506.0217	
	2.00	1.00	-356.66667*	162.13621	.040	-696.0217	-17.3117	
		3.00	-154.30000	170.04989	.376	-510.2185	201.6185	
		4.00	-190.00000	162.13621	.256	-529.3550	149.3550	
	3.00	1.00	-202.36667	170.04989	.249	-558.2852	153.5519	
		2.00	154.30000	170.04989	.376	-201.6185	510.2185	
		4.00	-35.70000	170.04989	.836	-391.6185	320.2185	
	4.00	1.00	-166.66667	162.13621	.317	-506.0217	172.6883	
		2.00	190.00000	162.13621	.256	-149.3550	529.3550	
		3.00	35.70000	170.04989	.836	-320.2185	391.6185	
Time90min	1.00	2.00	144.83333	115.17915	.224	-96.2394	385.9061	
		3.00	83.76667	120.80091	.496	-169.0725	336.6059	
		4.00	-.96667	115.17915	.993	-242.0394	240.1061	
	2.00	1.00	-144.83333	115.17915	.224	-385.9061	96.2394	
		3.00	-61.06667	120.80091	.619	-313.9059	191.7725	
		4.00	-145.80000	115.17915	.221	-386.8727	95.2727	
	3.00	1.00	-83.76667	120.80091	.496	-336.6059	169.0725	
		2.00	61.06667	120.80091	.619	-191.7725	313.9059	
		4.00	-84.73333	120.80091	.492	-337.5725	168.1059	
	4.00	1.00	.96667	115.17915	.993	-240.1061	242.0394	
		2.00	145.80000	115.17915	.221	-95.2727	386.8727	
		3.00	84.73333	120.80091	.492	-168.1059	337.5725	
Time100min	1.00	2.00	323.83333*	112.30349	.010	88.7794	558.8872	
		3.00	241.46667	117.78489	.054	-5.0599	487.9933	
		4.00	110.26667	112.30349	.339	-124.7872	345.3206	
	2.00	1.00	-323.83333*	112.30349	.010	-558.8872	-88.7794	
		3.00	-82.36667	117.78489	.493	-328.8933	164.1599	
		4.00	-213.56667	112.30349	.072	-448.6206	21.4872	
	3.00	1.00	-241.46667	117.78489	.054	-487.9933	5.0599	
		2.00	82.36667	117.78489	.493	-164.1599	328.8933	
		4.00	-131.20000	117.78489	.279	-377.7266	115.3266	
	4.00	1.00	-110.26667	112.30349	.339	-345.3206	124.7872	
		2.00	213.56667	112.30349	.072	-21.4872	448.6206	
		3.00	131.20000	117.78489	.279	-115.3266	377.7266	
Time110min	1.00	2.00	248.16667*	93.26929	.015	52.9518	443.3815	
		3.00	231.73333*	97.82166	.029	26.9902	436.4764	
		4.00	109.13333	93.26929	.256	-86.0815	304.3482	
	2.00	1.00	-248.16667*	93.26929	.015	-443.3815	-52.9518	
		3.00	-16.43333	97.82166	.868	-221.1764	188.3098	
		4.00	-139.03333	93.26929	.152	-334.2482	56.1815	
	3.00	1.00	-231.73333*	97.82166	.029	-436.4764	-26.9902	
		2.00	16.43333	97.82166	.868	-188.3098	221.1764	
		4.00	-122.60000	97.82166	.225	-327.3431	82.1431	
	4.00	1.00	-109.13333	93.26929	.256	-304.3482	86.0815	
		2.00	139.03333	93.26929	.152	-56.1815	334.2482	
		3.00	122.60000	97.82166	.225	-82.1431	327.3431	
Time120min	1.00	2.00	391.00000*	80.01232	.000	223.5323	558.4677	
		3.00	271.10000*	83.91763	.004	95.4584	446.7416	
		4.00	114.03333	80.01232	.170	-53.4344	281.5010	
	2.00	1.00	-391.00000*	80.01232	.000	-558.4677	-223.5323	
		3.00	-119.90000	83.91763	.169	-295.5416	55.7416	
		4.00	-276.96667*	80.01232	.003	-444.4344	-109.4990	
	3.00	1.00	-271.10000*	83.91763	.004	-446.7416	-95.4584	
		2.00	119.90000	83.91763	.169	-55.7416	295.5416	
		4.00	-157.06667	83.91763	.077	-332.7083	18.5749	
	4.00	1.00	-114.03333	80.01232	.170	-281.5010	53.4344	
		2.00	276.96667*	80.01232	.003	109.4990	444.4344	
		3.00	157.06667	83.91763	.077	-18.5749	332.7083	

*. §¡­È®tªº显µÛ©Ê¤ô¥­为 0.05¡C	
