# Supplementary material for: Taxifolin protects rat against myocardial ischemia/reperfusion injury by modulating the mitochondrial apoptosis pathway
Source: PeerJ. 2019 Jan 31;7:e6383. doi: 10.7717/peerj.6383 (PMC6360081; doi:10.7717/peerj.6383)
Supplement: Supplemental Information 6 [file peerj-07-6383-s006.zip › Statistical Reporting/Analysis results/Word file form/GSH-PX.doc]

ONEWAY GSHPX BY Group
  /STATISTICS HOMOGENEITY
  /MISSING ANALYSIS
  /POSTHOC=LSD ALPHA(0.05).

Oneway

C:\Users\Administrator\Desktop\Statistical Reporting\GSH-PX.sav

Test of Homogeneity of Variances	
GSHPX  	
Levene Statistic	df1	df2	Sig.	
.145	3	21	.932	

ANOVA	
GSHPX  	
	Sun of Squares	df	Mean Square	F	Sig.	
Between Groups	328765.873	3	109588.624	22.445	.000	
Within Groups	102534.775	21	4882.608			
Total	431300.648	24				

Post Hoc Tests
Multiple Comparisons	
Dependent Variable: GSH-PX	
LSD  	
(I) Group	(J) Group	Mean Difference (I-J)	Std. Error	Sig.	95% Confidence interval	
					Lower Bound	Lower Bound	
1.00	2.00	283.22921*	37.73717	.000	204.7505	361.7080	
	3.00	174.61201*	42.31182	.000	86.6198	262.6042	
	4.00	57.26414	40.34273	.170	-26.6332	141.1614	
2.00	1.00	-283.22921*	37.73717	.000	-361.7080	-204.7505	
	3.00	-108.61720*	39.83526	.013	-191.4592	-25.7752	
	4.00	-225.96508*	37.73717	.000	-304.4438	-147.4863	
3.00	1.00	-174.61201*	42.31182	.000	-262.6042	-86.6198	
	2.00	108.61720*	39.83526	.013	25.7752	191.4592	
	4.00	-117.34787*	42.31182	.011	-205.3401	-29.3556	
4.00	1.00	-57.26414	40.34273	.170	-141.1614	26.6332	
	2.00	225.96508*	37.73717	.000	147.4863	304.4438	
	3.00	117.34787*	42.31182	.011	29.3556	205.3401	

*. The mean difference is significant at the 0.05 level.	
