# Supplementary material for: Taxifolin protects rat against myocardial ischemia/reperfusion injury by modulating the mitochondrial apoptosis pathway
Source: PeerJ. 2019 Jan 31;7:e6383. doi: 10.7717/peerj.6383 (PMC6360081; doi:10.7717/peerj.6383)
Supplement: Supplemental Information 6 [file peerj-07-6383-s006.zip › Statistical Reporting/Analysis results/Word file form/HR.doc]

ONEWAY Time10min Time20min Time30min Time60min Time70min Time80min Time90min Time100min Time110min Time120min BY Group
  /STATISTICS HOMOGENEITY
  /MISSING ANALYSIS
  /POSTHOC=LSD ALPHA(0.05).

Oneway

[�”�˜�W11] C:\Users\Administrator\Desktop\Statistical Reporting\HR.sav

¤è®t齐©Ê检验	
	Levene 统计¶q	df1	df2	显µÛ©Ê	
Time10min	2.325	3	19	.107	
Time20min	2.889	3	19	.062	
Time30min	4.093	3	19	.021	
Time60min	.572	3	19	.641	
Time70min	.365	3	19	.779	
Time80min	.029	3	19	.993	
Time90min	.829	3	19	.494	
Time100min	2.130	3	19	.130	
Time110min	2.186	3	19	.123	
Time120min	1.273	3	19	.312	

单¦]¯À¤è®t¤ÀªR	
	¥­¤è©M	df	§¡¤è	F	显µÛ©Ê	
Time10min	组间	2114.309	3	704.770	4.300	.018	
	组内	3114.300	19	163.911			
	总数	5228.609	22				
Time20min	组间	968.336	3	322.779	2.515	.089	
	组内	2438.533	19	128.344			
	总数	3406.870	22				
Time30min	组间	523.990	3	174.663	.694	.567	
	组内	4784.967	19	251.840			
	总数	5308.957	22				
Time60min	组间	2393.568	3	797.856	3.918	.025	
	组内	3868.867	19	203.625			
	总数	6262.435	22				
Time70min	组间	1076.235	3	358.745	2.007	.147	
	组内	3396.200	19	178.747			
	总数	4472.435	22				
Time80min	组间	529.867	3	176.622	1.396	.275	
	组内	2404.133	19	126.533			
	总数	2934.000	22				
Time90min	组间	1126.300	3	375.433	3.427	.038	
	组内	2081.700	19	109.563			
	总数	3208.000	22				
Time100min	组间	1398.671	3	466.224	3.827	.027	
	组内	2314.633	19	121.823			
	总数	3713.304	22				
Time110min	组间	1754.106	3	584.702	6.000	.005	
	组内	1851.633	19	97.454			
	总数	3605.739	22				
Time120min	组间	2414.568	3	804.856	6.945	.002	
	组内	2201.867	19	115.888			
	总数	4616.435	22				

Post Hoc Tests
¦h­«¤ñ较	
LSD  	
¦]变¶q	(I) Group	(J) Group	§¡­È®t (I-J)	标­ã误	显µÛ©Ê	95% ¸m«H区间	
						¤U­­	¤W­­	
Time10min	1.00	2.00	-7.66667	7.39167	.313	-23.1376	7.8043	
		3.00	-19.13333*	7.75245	.023	-35.3594	-2.9073	
		4.00	-24.16667*	7.39167	.004	-39.6376	-8.6957	
	2.00	1.00	7.66667	7.39167	.313	-7.8043	23.1376	
		3.00	-11.46667	7.75245	.156	-27.6927	4.7594	
		4.00	-16.50000*	7.39167	.038	-31.9710	-1.0290	
	3.00	1.00	19.13333*	7.75245	.023	2.9073	35.3594	
		2.00	11.46667	7.75245	.156	-4.7594	27.6927	
		4.00	-5.03333	7.75245	.524	-21.2594	11.1927	
	4.00	1.00	24.16667*	7.39167	.004	8.6957	39.6376	
		2.00	16.50000*	7.39167	.038	1.0290	31.9710	
		3.00	5.03333	7.75245	.524	-11.1927	21.2594	
Time20min	1.00	2.00	-4.33333	6.54074	.516	-18.0233	9.3566	
		3.00	1.60000	6.85999	.818	-12.7581	15.9581	
		4.00	-15.00000*	6.54074	.033	-28.6899	-1.3101	
	2.00	1.00	4.33333	6.54074	.516	-9.3566	18.0233	
		3.00	5.93333	6.85999	.398	-8.4248	20.2915	
		4.00	-10.66667	6.54074	.119	-24.3566	3.0233	
	3.00	1.00	-1.60000	6.85999	.818	-15.9581	12.7581	
		2.00	-5.93333	6.85999	.398	-20.2915	8.4248	
		4.00	-16.60000*	6.85999	.026	-30.9581	-2.2419	
	4.00	1.00	15.00000*	6.54074	.033	1.3101	28.6899	
		2.00	10.66667	6.54074	.119	-3.0233	24.3566	
		3.00	16.60000*	6.85999	.026	2.2419	30.9581	
Time30min	1.00	2.00	6.83333	9.16225	.465	-12.3435	26.0101	
		3.00	1.13333	9.60945	.907	-18.9795	21.2461	
		4.00	-6.33333	9.16225	.498	-25.5101	12.8435	
	2.00	1.00	-6.83333	9.16225	.465	-26.0101	12.3435	
		3.00	-5.70000	9.60945	.560	-25.8128	14.4128	
		4.00	-13.16667	9.16225	.167	-32.3435	6.0101	
	3.00	1.00	-1.13333	9.60945	.907	-21.2461	18.9795	
		2.00	5.70000	9.60945	.560	-14.4128	25.8128	
		4.00	-7.46667	9.60945	.447	-27.5795	12.6461	
	4.00	1.00	6.33333	9.16225	.498	-12.8435	25.5101	
		2.00	13.16667	9.16225	.167	-6.0101	32.3435	
		3.00	7.46667	9.60945	.447	-12.6461	27.5795	
Time60min	1.00	2.00	18.16667*	8.23862	.040	.9230	35.4103	
		3.00	7.26667	8.64074	.411	-10.8186	25.3519	
		4.00	-9.16667	8.23862	.280	-26.4103	8.0770	
	2.00	1.00	-18.16667*	8.23862	.040	-35.4103	-.9230	
		3.00	-10.90000	8.64074	.222	-28.9853	7.1853	
		4.00	-27.33333*	8.23862	.004	-44.5770	-10.0897	
	3.00	1.00	-7.26667	8.64074	.411	-25.3519	10.8186	
		2.00	10.90000	8.64074	.222	-7.1853	28.9853	
		4.00	-16.43333	8.64074	.072	-34.5186	1.6519	
	4.00	1.00	9.16667	8.23862	.280	-8.0770	26.4103	
		2.00	27.33333*	8.23862	.004	10.0897	44.5770	
		3.00	16.43333	8.64074	.072	-1.6519	34.5186	
Time70min	1.00	2.00	12.33333	7.71897	.127	-3.8227	28.4893	
		3.00	3.76667	8.09572	.647	-13.1779	20.7112	
		4.00	-6.16667	7.71897	.434	-22.3227	9.9893	
	2.00	1.00	-12.33333	7.71897	.127	-28.4893	3.8227	
		3.00	-8.56667	8.09572	.303	-25.5112	8.3779	
		4.00	-18.50000*	7.71897	.027	-34.6560	-2.3440	
	3.00	1.00	-3.76667	8.09572	.647	-20.7112	13.1779	
		2.00	8.56667	8.09572	.303	-8.3779	25.5112	
		4.00	-9.93333	8.09572	.235	-26.8779	7.0112	
	4.00	1.00	6.16667	7.71897	.434	-9.9893	22.3227	
		2.00	18.50000*	7.71897	.027	2.3440	34.6560	
		3.00	9.93333	8.09572	.235	-7.0112	26.8779	
Time80min	1.00	2.00	13.00000	6.49444	.060	-.5930	26.5930	
		3.00	6.80000	6.81143	.331	-7.4565	21.0565	
		4.00	4.33333	6.49444	.513	-9.2597	17.9264	
	2.00	1.00	-13.00000	6.49444	.060	-26.5930	.5930	
		3.00	-6.20000	6.81143	.374	-20.4565	8.0565	
		4.00	-8.66667	6.49444	.198	-22.2597	4.9264	
	3.00	1.00	-6.80000	6.81143	.331	-21.0565	7.4565	
		2.00	6.20000	6.81143	.374	-8.0565	20.4565	
		4.00	-2.46667	6.81143	.721	-16.7231	11.7898	
	4.00	1.00	-4.33333	6.49444	.513	-17.9264	9.2597	
		2.00	8.66667	6.49444	.198	-4.9264	22.2597	
		3.00	2.46667	6.81143	.721	-11.7898	16.7231	
Time90min	1.00	2.00	19.00000*	6.04327	.005	6.3513	31.6487	
		3.00	12.76667	6.33823	.058	-.4994	26.0327	
		4.00	9.33333	6.04327	.139	-3.3154	21.9820	
	2.00	1.00	-19.00000*	6.04327	.005	-31.6487	-6.3513	
		3.00	-6.23333	6.33823	.338	-19.4994	7.0327	
		4.00	-9.66667	6.04327	.126	-22.3154	2.9820	
	3.00	1.00	-12.76667	6.33823	.058	-26.0327	.4994	
		2.00	6.23333	6.33823	.338	-7.0327	19.4994	
		4.00	-3.43333	6.33823	.594	-16.6994	9.8327	
	4.00	1.00	-9.33333	6.04327	.139	-21.9820	3.3154	
		2.00	9.66667	6.04327	.126	-2.9820	22.3154	
		3.00	3.43333	6.33823	.594	-9.8327	16.6994	
Time100min	1.00	2.00	21.00000*	6.37241	.004	7.6624	34.3376	
		3.00	11.30000	6.68344	.107	-2.6886	25.2886	
		4.00	6.66667	6.37241	.309	-6.6709	20.0043	
	2.00	1.00	-21.00000*	6.37241	.004	-34.3376	-7.6624	
		3.00	-9.70000	6.68344	.163	-23.6886	4.2886	
		4.00	-14.33333*	6.37241	.037	-27.6709	-.9957	
	3.00	1.00	-11.30000	6.68344	.107	-25.2886	2.6886	
		2.00	9.70000	6.68344	.163	-4.2886	23.6886	
		4.00	-4.63333	6.68344	.497	-18.6219	9.3553	
	4.00	1.00	-6.66667	6.37241	.309	-20.0043	6.6709	
		2.00	14.33333*	6.37241	.037	.9957	27.6709	
		3.00	4.63333	6.68344	.497	-9.3553	18.6219	
Time110min	1.00	2.00	23.50000*	5.69954	.001	11.5707	35.4293	
		3.00	9.70000	5.97773	.121	-2.8115	22.2115	
		4.00	6.83333	5.69954	.245	-5.0959	18.7626	
	2.00	1.00	-23.50000*	5.69954	.001	-35.4293	-11.5707	
		3.00	-13.80000*	5.97773	.032	-26.3115	-1.2885	
		4.00	-16.66667*	5.69954	.009	-28.5959	-4.7374	
	3.00	1.00	-9.70000	5.97773	.121	-22.2115	2.8115	
		2.00	13.80000*	5.97773	.032	1.2885	26.3115	
		4.00	-2.86667	5.97773	.637	-15.3782	9.6449	
	4.00	1.00	-6.83333	5.69954	.245	-18.7626	5.0959	
		2.00	16.66667*	5.69954	.009	4.7374	28.5959	
		3.00	2.86667	5.97773	.637	-9.6449	15.3782	
Time120min	1.00	2.00	27.33333*	6.21524	.000	14.3247	40.3420	
		3.00	14.40000*	6.51860	.040	.7564	28.0436	
		4.00	7.66667	6.21524	.232	-5.3420	20.6753	
	2.00	1.00	-27.33333*	6.21524	.000	-40.3420	-14.3247	
		3.00	-12.93333	6.51860	.062	-26.5769	.7103	
		4.00	-19.66667*	6.21524	.005	-32.6753	-6.6580	
	3.00	1.00	-14.40000*	6.51860	.040	-28.0436	-.7564	
		2.00	12.93333	6.51860	.062	-.7103	26.5769	
		4.00	-6.73333	6.51860	.315	-20.3769	6.9103	
	4.00	1.00	-7.66667	6.21524	.232	-20.6753	5.3420	
		2.00	19.66667*	6.21524	.005	6.6580	32.6753	
		3.00	6.73333	6.51860	.315	-6.9103	20.3769	

*. §¡­È®tªº显µÛ©Ê¤ô¥­为 0.05¡C	
