# Supplementary material for: Taxifolin protects rat against myocardial ischemia/reperfusion injury by modulating the mitochondrial apoptosis pathway
Source: PeerJ. 2019 Jan 31;7:e6383. doi: 10.7717/peerj.6383 (PMC6360081; doi:10.7717/peerj.6383)
Supplement: Supplemental Information 6 [file peerj-07-6383-s006.zip › Statistical Reporting/Analysis results/Word file form/LDH.doc]

ONEWAY Time25min Time63min Time90min Time120min BY Group
  /STATISTICS HOMOGENEITY
  /MISSING ANALYSIS
  /POSTHOC=LSD ALPHA(0.05).

Oneway

C:\Users\Administrator\Desktop\Statistical Reporting\LDH.sav

Test of Homogeneity of Variances	
	Levene Statistic	df1	df2	Sig.	
Time25min	.847	3	19	.485	
Time63min	.989	3	19	.419	
Time90min	.904	3	19	.457	
Time120min	.229	3	19	.875	

ANOVA	
	Sun of Squares	df	Mean Square	F	Sig.	
Time25min	Between Groups	232.972	3	77.657	.571	.641	
	Within Groups	2581.951	19	135.892			
	Total	2814.923	22				
Time63min	Between Groups	422.163	3	140.721	1.298	.304	
	Within Groups	2060.616	19	108.453			
	Total	2482.779	22				
Time90min	Between Groups	3965.196	3	1321.732	10.329	.000	
	Within Groups	2431.376	19	127.967			
	Total	6396.572	22				
Time120min	Between Groups	3752.169	3	1250.723	10.291	.000	
	Within Groups	2309.247	19	121.539			
	Total	6061.416	22				

Post Hoc Tests
Multiple Comparisons	
LSD  	
Dependent Variable	(I) Group	(J) Group	Mean Difference (I-J)	Std. Error	Sig.	95% Confidence interval	
						Lower bound	Upper Bound	
Time25min	1.00	2.00	8.64333	6.73033	.214	-5.4434	22.7301	
		3.00	5.70800	7.05883	.429	-9.0663	20.4823	
		4.00	4.04667	6.73033	.555	-10.0401	18.1334	
	2.00	1.00	-8.64333	6.73033	.214	-22.7301	5.4434	
		3.00	-2.93533	7.05883	.682	-17.7096	11.8390	
		4.00	-4.59667	6.73033	.503	-18.6834	9.4901	
	3.00	1.00	-5.70800	7.05883	.429	-20.4823	9.0663	
		2.00	2.93533	7.05883	.682	-11.8390	17.7096	
		4.00	-1.66133	7.05883	.816	-16.4356	13.1130	
	4.00	1.00	-4.04667	6.73033	.555	-18.1334	10.0401	
		2.00	4.59667	6.73033	.503	-9.4901	18.6834	
		3.00	1.66133	7.05883	.816	-13.1130	16.4356	
Time63min	1.00	2.00	-9.21624	6.01258	.142	-21.8007	3.3682	
		3.00	-11.47291	6.30605	.085	-24.6716	1.7258	
		4.00	-7.10690	6.01258	.252	-19.6914	5.4776	
	2.00	1.00	9.21624	6.01258	.142	-3.3682	21.8007	
		3.00	-2.25667	6.30605	.724	-15.4554	10.9420	
		4.00	2.10935	6.01258	.730	-10.4751	14.6938	
	3.00	1.00	11.47291	6.30605	.085	-1.7258	24.6716	
		2.00	2.25667	6.30605	.724	-10.9420	15.4554	
		4.00	4.36602	6.30605	.497	-8.8327	17.5647	
	4.00	1.00	7.10690	6.01258	.252	-5.4776	19.6914	
		2.00	-2.10935	6.01258	.730	-14.6938	10.4751	
		3.00	-4.36602	6.30605	.497	-17.5647	8.8327	
Time90min	1.00	2.00	-35.66667*	6.53114	.000	-49.3365	-21.9968	
		3.00	-24.24233*	6.84991	.002	-38.5794	-9.9053	
		4.00	-18.78167*	6.53114	.010	-32.4515	-5.1118	
	2.00	1.00	35.66667*	6.53114	.000	21.9968	49.3365	
		3.00	11.42433	6.84991	.112	-2.9127	25.7614	
		4.00	16.88500*	6.53114	.018	3.2152	30.5548	
	3.00	1.00	24.24233*	6.84991	.002	9.9053	38.5794	
		2.00	-11.42433	6.84991	.112	-25.7614	2.9127	
		4.00	5.46067	6.84991	.435	-8.8764	19.7977	
	4.00	1.00	18.78167*	6.53114	.010	5.1118	32.4515	
		2.00	-16.88500*	6.53114	.018	-30.5548	-3.2152	
		3.00	-5.46067	6.84991	.435	-19.7977	8.8764	
Time120min	1.00	2.00	-34.19873*	6.36499	.000	-47.5208	-20.8767	
		3.00	-18.33940*	6.67566	.013	-32.3117	-4.3671	
		4.00	-10.13707	6.36499	.128	-23.4591	3.1850	
	2.00	1.00	34.19873*	6.36499	.000	20.8767	47.5208	
		3.00	15.85933*	6.67566	.028	1.8870	29.8316	
		4.00	24.06167*	6.36499	.001	10.7396	37.3837	
	3.00	1.00	18.33940*	6.67566	.013	4.3671	32.3117	
		2.00	-15.85933*	6.67566	.028	-29.8316	-1.8870	
		4.00	8.20233	6.67566	.234	-5.7700	22.1746	
	4.00	1.00	10.13707	6.36499	.128	-3.1850	23.4591	
		2.00	-24.06167*	6.36499	.001	-37.3837	-10.7396	
		3.00	-8.20233	6.67566	.234	-22.1746	5.7700	

*. The mean difference is significant at the 0.05 level.	
