# Supplementary material for: Taxifolin protects rat against myocardial ischemia/reperfusion injury by modulating the mitochondrial apoptosis pathway
Source: PeerJ. 2019 Jan 31;7:e6383. doi: 10.7717/peerj.6383 (PMC6360081; doi:10.7717/peerj.6383)
Supplement: Supplemental Information 6 [file peerj-07-6383-s006.zip › Statistical Reporting/Analysis results/Word file form/LVDP.doc]

ONEWAY Time10min Time20min Time30min Time60min Time70min Time80min Time90min Time100min Time110min Time120min BY Group
  /STATISTICS HOMOGENEITY
  /MISSING ANALYSIS
  /POSTHOC=LSD ALPHA(0.05).

Oneway

¤è®t齐©Ê检验	
	Levene 统计¶q	df1	df2	显µÛ©Ê	
Time10min	.906	3	19	.457	
Time20min	.447	3	19	.722	
Time30min	.554	3	19	.652	
Time60min	.995	3	19	.417	
Time70min	1.772	3	19	.187	
Time80min	1.948	3	19	.156	
Time90min	1.480	3	19	.252	
Time100min	2.843	3	19	.065	
Time110min	1.761	3	19	.189	
Time120min	1.822	3	19	.177	

单¦]¯À¤è®t¤ÀªR	
	¥­¤è©M	df	§¡¤è	F	显µÛ©Ê	
Time10min	组间	209.446	3	69.815	.637	.600	
	组内	2082.467	19	109.604			
	总数	2291.913	22				
Time20min	组间	151.590	3	50.530	.444	.724	
	组内	2161.367	19	113.756			
	总数	2312.957	22				
Time30min	组间	88.026	3	29.342	.256	.856	
	组内	2175.800	19	114.516			
	总数	2263.826	22				
Time60min	组间	621.959	3	207.320	1.683	.204	
	组内	2339.867	19	123.151			
	总数	2961.826	22				
Time70min	组间	1034.670	3	344.890	4.050	.022	
	组内	1618.200	19	85.168			
	总数	2652.870	22				
Time80min	组间	747.751	3	249.250	2.432	.097	
	组内	1947.467	19	102.498			
	总数	2695.217	22				
Time90min	组间	1373.445	3	457.815	5.028	.010	
	组内	1730.033	19	91.054			
	总数	3103.478	22				
Time100min	组间	1448.178	3	482.726	5.982	.005	
	组内	1533.300	19	80.700			
	总数	2981.478	22				
Time110min	组间	1798.403	3	599.468	6.722	.003	
	组内	1694.467	19	89.182			
	总数	3492.870	22				
Time120min	组间	2080.135	3	693.378	6.804	.003	
	组内	1936.300	19	101.911			
	总数	4016.435	22				

Post Hoc Tests
¦h­«¤ñ较	
LSD  	
¦]变¶q	(I) Group	(J) Group	§¡­È®t (I-J)	标­ã误	显µÛ©Ê	95% ¸m«H区间	
						¤U­­	¤W­­	
Time10min	1.00	2.00	5.16667	6.04438	.403	-7.4844	17.8177	
		3.00	-2.70000	6.33940	.675	-15.9685	10.5685	
		4.00	3.66667	6.04438	.551	-8.9844	16.3177	
	2.00	1.00	-5.16667	6.04438	.403	-17.8177	7.4844	
		3.00	-7.86667	6.33940	.230	-21.1352	5.4018	
		4.00	-1.50000	6.04438	.807	-14.1510	11.1510	
	3.00	1.00	2.70000	6.33940	.675	-10.5685	15.9685	
		2.00	7.86667	6.33940	.230	-5.4018	21.1352	
		4.00	6.36667	6.33940	.328	-6.9018	19.6352	
	4.00	1.00	-3.66667	6.04438	.551	-16.3177	8.9844	
		2.00	1.50000	6.04438	.807	-11.1510	14.1510	
		3.00	-6.36667	6.33940	.328	-19.6352	6.9018	
Time20min	1.00	2.00	2.83333	6.15782	.651	-10.0551	15.7218	
		3.00	-3.73333	6.45837	.570	-17.2509	9.7842	
		4.00	2.66667	6.15782	.670	-10.2218	15.5551	
	2.00	1.00	-2.83333	6.15782	.651	-15.7218	10.0551	
		3.00	-6.56667	6.45837	.322	-20.0842	6.9509	
		4.00	-.16667	6.15782	.979	-13.0551	12.7218	
	3.00	1.00	3.73333	6.45837	.570	-9.7842	17.2509	
		2.00	6.56667	6.45837	.322	-6.9509	20.0842	
		4.00	6.40000	6.45837	.334	-7.1175	19.9175	
	4.00	1.00	-2.66667	6.15782	.670	-15.5551	10.2218	
		2.00	.16667	6.15782	.979	-12.7218	13.0551	
		3.00	-6.40000	6.45837	.334	-19.9175	7.1175	
Time30min	1.00	2.00	2.50000	6.17834	.690	-10.4314	15.4314	
		3.00	-2.86667	6.47990	.663	-16.4293	10.6959	
		4.00	1.50000	6.17834	.811	-11.4314	14.4314	
	2.00	1.00	-2.50000	6.17834	.690	-15.4314	10.4314	
		3.00	-5.36667	6.47990	.418	-18.9293	8.1959	
		4.00	-1.00000	6.17834	.873	-13.9314	11.9314	
	3.00	1.00	2.86667	6.47990	.663	-10.6959	16.4293	
		2.00	5.36667	6.47990	.418	-8.1959	18.9293	
		4.00	4.36667	6.47990	.509	-9.1959	17.9293	
	4.00	1.00	-1.50000	6.17834	.811	-14.4314	11.4314	
		2.00	1.00000	6.17834	.873	-11.9314	13.9314	
		3.00	-4.36667	6.47990	.509	-17.9293	9.1959	
Time60min	1.00	2.00	11.33333	6.40705	.093	-2.0768	24.7434	
		3.00	7.60000	6.71977	.272	-6.4646	21.6646	
		4.00	13.33333	6.40705	.051	-.0768	26.7434	
	2.00	1.00	-11.33333	6.40705	.093	-24.7434	2.0768	
		3.00	-3.73333	6.71977	.585	-17.7980	10.3313	
		4.00	2.00000	6.40705	.758	-11.4101	15.4101	
	3.00	1.00	-7.60000	6.71977	.272	-21.6646	6.4646	
		2.00	3.73333	6.71977	.585	-10.3313	17.7980	
		4.00	5.73333	6.71977	.404	-8.3313	19.7980	
	4.00	1.00	-13.33333	6.40705	.051	-26.7434	.0768	
		2.00	-2.00000	6.40705	.758	-15.4101	11.4101	
		3.00	-5.73333	6.71977	.404	-19.7980	8.3313	
Time70min	1.00	2.00	18.50000*	5.32818	.003	7.3480	29.6520	
		3.00	10.56667	5.58824	.074	-1.1297	22.2630	
		4.00	9.00000	5.32818	.108	-2.1520	20.1520	
	2.00	1.00	-18.50000*	5.32818	.003	-29.6520	-7.3480	
		3.00	-7.93333	5.58824	.172	-19.6297	3.7630	
		4.00	-9.50000	5.32818	.091	-20.6520	1.6520	
	3.00	1.00	-10.56667	5.58824	.074	-22.2630	1.1297	
		2.00	7.93333	5.58824	.172	-3.7630	19.6297	
		4.00	-1.56667	5.58824	.782	-13.2630	10.1297	
	4.00	1.00	-9.00000	5.32818	.108	-20.1520	2.1520	
		2.00	9.50000	5.32818	.091	-1.6520	20.6520	
		3.00	1.56667	5.58824	.782	-10.1297	13.2630	
Time80min	1.00	2.00	15.33333*	5.84518	.017	3.0992	27.5674	
		3.00	10.46667	6.13047	.104	-2.3646	23.2979	
		4.00	6.66667	5.84518	.268	-5.5674	18.9008	
	2.00	1.00	-15.33333*	5.84518	.017	-27.5674	-3.0992	
		3.00	-4.86667	6.13047	.437	-17.6979	7.9646	
		4.00	-8.66667	5.84518	.155	-20.9008	3.5674	
	3.00	1.00	-10.46667	6.13047	.104	-23.2979	2.3646	
		2.00	4.86667	6.13047	.437	-7.9646	17.6979	
		4.00	-3.80000	6.13047	.543	-16.6312	9.0312	
	4.00	1.00	-6.66667	5.84518	.268	-18.9008	5.5674	
		2.00	8.66667	5.84518	.155	-3.5674	20.9008	
		3.00	3.80000	6.13047	.543	-9.0312	16.6312	
Time90min	1.00	2.00	20.00000*	5.50922	.002	8.4691	31.5309	
		3.00	16.60000*	5.77811	.010	4.5063	28.6937	
		4.00	9.83333	5.50922	.090	-1.6976	21.3643	
	2.00	1.00	-20.00000*	5.50922	.002	-31.5309	-8.4691	
		3.00	-3.40000	5.77811	.563	-15.4937	8.6937	
		4.00	-10.16667	5.50922	.081	-21.6976	1.3643	
	3.00	1.00	-16.60000*	5.77811	.010	-28.6937	-4.5063	
		2.00	3.40000	5.77811	.563	-8.6937	15.4937	
		4.00	-6.76667	5.77811	.256	-18.8604	5.3271	
	4.00	1.00	-9.83333	5.50922	.090	-21.3643	1.6976	
		2.00	10.16667	5.50922	.081	-1.3643	21.6976	
		3.00	6.76667	5.77811	.256	-5.3271	18.8604	
Time100min	1.00	2.00	21.33333*	5.18652	.001	10.4778	32.1888	
		3.00	15.36667*	5.43967	.011	3.9813	26.7520	
		4.00	11.00000*	5.18652	.047	.1445	21.8555	
	2.00	1.00	-21.33333*	5.18652	.001	-32.1888	-10.4778	
		3.00	-5.96667	5.43967	.286	-17.3520	5.4187	
		4.00	-10.33333	5.18652	.061	-21.1888	.5222	
	3.00	1.00	-15.36667*	5.43967	.011	-26.7520	-3.9813	
		2.00	5.96667	5.43967	.286	-5.4187	17.3520	
		4.00	-4.36667	5.43967	.432	-15.7520	7.0187	
	4.00	1.00	-11.00000*	5.18652	.047	-21.8555	-.1445	
		2.00	10.33333	5.18652	.061	-.5222	21.1888	
		3.00	4.36667	5.43967	.432	-7.0187	15.7520	
Time110min	1.00	2.00	22.83333*	5.45229	.000	11.4216	34.2451	
		3.00	19.53333*	5.71841	.003	7.5646	31.5021	
		4.00	13.16667*	5.45229	.026	1.7549	24.5784	
	2.00	1.00	-22.83333*	5.45229	.000	-34.2451	-11.4216	
		3.00	-3.30000	5.71841	.571	-15.2688	8.6688	
		4.00	-9.66667	5.45229	.092	-21.0784	1.7451	
	3.00	1.00	-19.53333*	5.71841	.003	-31.5021	-7.5646	
		2.00	3.30000	5.71841	.571	-8.6688	15.2688	
		4.00	-6.36667	5.71841	.279	-18.3354	5.6021	
	4.00	1.00	-13.16667*	5.45229	.026	-24.5784	-1.7549	
		2.00	9.66667	5.45229	.092	-1.7451	21.0784	
		3.00	6.36667	5.71841	.279	-5.6021	18.3354	
Time120min	1.00	2.00	24.66667*	5.82839	.000	12.4677	36.8656	
		3.00	18.13333*	6.11287	.008	5.3389	30.9277	
		4.00	8.50000	5.82839	.161	-3.6990	20.6990	
	2.00	1.00	-24.66667*	5.82839	.000	-36.8656	-12.4677	
		3.00	-6.53333	6.11287	.299	-19.3277	6.2611	
		4.00	-16.16667*	5.82839	.012	-28.3656	-3.9677	
	3.00	1.00	-18.13333*	6.11287	.008	-30.9277	-5.3389	
		2.00	6.53333	6.11287	.299	-6.2611	19.3277	
		4.00	-9.63333	6.11287	.132	-22.4277	3.1611	
	4.00	1.00	-8.50000	5.82839	.161	-20.6990	3.6990	
		2.00	16.16667*	5.82839	.012	3.9677	28.3656	
		3.00	9.63333	6.11287	.132	-3.1611	22.4277	

*. §¡­È®tªº显µÛ©Ê¤ô¥­为 0.05¡C	
