# Supplementary material for: Taxifolin protects rat against myocardial ischemia/reperfusion injury by modulating the mitochondrial apoptosis pathway
Source: PeerJ. 2019 Jan 31;7:e6383. doi: 10.7717/peerj.6383 (PMC6360081; doi:10.7717/peerj.6383)
Supplement: Supplemental Information 6 [file peerj-07-6383-s006.zip › Statistical Reporting/Analysis results/Word file form/MDA.doc]

ONEWAY MDA BY Group
  /STATISTICS HOMOGENEITY
  /MISSING ANALYSIS
  /POSTHOC=LSD ALPHA(0.05).

Oneway

C:\Users\Administrator\Desktop\Statistical Reporting\MDA.sav

Test of Homogeneity of Variances	
MDA  	
Levene Statistic	df1	df2	Sig.	
1.612	3	22	.215	

ANOVA	
MDA  	
	Sun of Squares	df	Mean Square	F	Sig.	
Between Groups	1240.071	3	413.357	15.437	.000	
Within Groups	589.084	22	26.777			
Total	1829.156	25				

Post Hoc Tests
Multiple Comparisons	
Dependent Variable: MDA	
LSD  	
(I) Group	(J) Group	Mean Difference (I-J)	Std. Error	Sig.	95% Confidence interval	
					Lower Bound	Lower Bound	
1.00	2.00	-16.72760*	2.79461	.000	-22.5233	-10.9319	
	3.00	-4.95103	3.13338	.128	-11.4493	1.5472	
	4.00	-2.05436	2.87889	.483	-8.0248	3.9161	
2.00	1.00	16.72760*	2.79461	.000	10.9319	22.5233	
	3.00	11.77657*	2.94998	.001	5.6587	17.8945	
	4.00	14.67324*	2.67811	.000	9.1192	20.2273	
3.00	1.00	4.95103	3.13338	.128	-1.5472	11.4493	
	2.00	-11.77657*	2.94998	.001	-17.8945	-5.6587	
	4.00	2.89667	3.02994	.349	-3.3870	9.1804	
4.00	1.00	2.05436	2.87889	.483	-3.9161	8.0248	
	2.00	-14.67324*	2.67811	.000	-20.2273	-9.1192	
	3.00	-2.89667	3.02994	.349	-9.1804	3.3870	

*. The mean difference is significant at the 0.05 level.	
