# Supplementary material for: Taxifolin protects rat against myocardial ischemia/reperfusion injury by modulating the mitochondrial apoptosis pathway
Source: PeerJ. 2019 Jan 31;7:e6383. doi: 10.7717/peerj.6383 (PMC6360081; doi:10.7717/peerj.6383)
Supplement: Supplemental Information 6 [file peerj-07-6383-s006.zip › Statistical Reporting/Analysis results/Word file form/RPP.doc]

ONEWAY Time10min Time20min Time30min Time60min Time70min Time80min Time90min Time100min Time110min Time120min BY Group
  /STATISTICS HOMOGENEITY
  /MISSING ANALYSIS
  /POSTHOC=LSD ALPHA(0.05).

Oneway

¤è®t齐©Ê检验	
	Levene 统计¶q	df1	df2	显µÛ©Ê	
Time10min	.761	3	19	.530	
Time20min	.426	3	19	.737	
Time30min	.417	3	19	.742	
Time60min	.340	3	19	.797	
Time70min	.981	3	19	.422	
Time80min	1.341	3	19	.291	
Time90min	1.257	3	19	.317	
Time100min	2.495	3	19	.091	
Time110min	.747	3	19	.537	
Time120min	1.048	3	19	.394	

单¦]¯À¤è®t¤ÀªR	
	¥­¤è©M	df	§¡¤è	F	显µÛ©Ê	
Time10min	组间	16932945.546	3	5644315.182	.939	.441	
	组内	114229684.367	19	6012088.651			
	总数	131162629.913	22				
Time20min	组间	3583967.439	3	1194655.813	.192	.901	
	组内	118238466.300	19	6223077.174			
	总数	121822433.739	22				
Time30min	组间	8167547.503	3	2722515.834	.296	.828	
	组内	174890703.367	19	9204773.861			
	总数	183058250.870	22				
Time60min	组间	31720248.217	3	10573416.072	1.219	.330	
	组内	164826123.000	19	8675059.105			
	总数	196546371.217	22				
Time70min	组间	83347563.746	3	27782521.249	4.350	.017	
	组内	121342100.167	19	6386426.325			
	总数	204689663.913	22				
Time80min	组间	58979859.184	3	19659953.061	3.156	.049	
	组内	118350346.033	19	6228965.581			
	总数	177330205.217	22				
Time90min	组间	116301461.457	3	38767153.819	7.429	.002	
	组内	99148535.500	19	5218343.974			
	总数	215449996.957	22				
Time100min	组间	118832511.312	3	39610837.104	9.134	.001	
	组内	82396814.167	19	4336674.430			
	总数	201229325.478	22				
Time110min	组间	142638508.459	3	47546169.486	9.087	.001	
	组内	99418885.367	19	5232572.914			
	总数	242057393.826	22				
Time120min	组间	171250480.206	3	57083493.402	9.838	.000	
	组内	110248431.533	19	5802549.028			
	总数	281498911.739	22				

Post Hoc Tests
¦h­«¤ñ较	
LSD  	
¦]变¶q	(I) Group	(J) Group	§¡­È®t (I-J)	标­ã误	显µÛ©Ê	95% ¸m«H区间	
						¤U­­	¤W­­	
Time10min	1.00	2.00	484.66667	1415.63751	.736	-2478.2967	3447.6300	
		3.00	-1804.56667	1484.73314	.239	-4912.1488	1303.0155	
		4.00	-929.66667	1415.63751	.519	-3892.6300	2033.2967	
	2.00	1.00	-484.66667	1415.63751	.736	-3447.6300	2478.2967	
		3.00	-2289.23333	1484.73314	.140	-5396.8155	818.3488	
		4.00	-1414.33333	1415.63751	.330	-4377.2967	1548.6300	
	3.00	1.00	1804.56667	1484.73314	.239	-1303.0155	4912.1488	
		2.00	2289.23333	1484.73314	.140	-818.3488	5396.8155	
		4.00	874.90000	1484.73314	.563	-2232.6822	3982.4822	
	4.00	1.00	929.66667	1415.63751	.519	-2033.2967	3892.6300	
		2.00	1414.33333	1415.63751	.330	-1548.6300	4377.2967	
		3.00	-874.90000	1484.73314	.563	-3982.4822	2232.6822	
Time20min	1.00	2.00	242.16667	1440.26354	.868	-2772.3396	3256.6729	
		3.00	-623.46667	1510.56114	.684	-3785.1075	2538.1741	
		4.00	-670.00000	1440.26354	.647	-3684.5062	2344.5062	
	2.00	1.00	-242.16667	1440.26354	.868	-3256.6729	2772.3396	
		3.00	-865.63333	1510.56114	.573	-4027.2741	2296.0075	
		4.00	-912.16667	1440.26354	.534	-3926.6729	2102.3396	
	3.00	1.00	623.46667	1510.56114	.684	-2538.1741	3785.1075	
		2.00	865.63333	1510.56114	.573	-2296.0075	4027.2741	
		4.00	-46.53333	1510.56114	.976	-3208.1741	3115.1075	
	4.00	1.00	670.00000	1440.26354	.647	-2344.5062	3684.5062	
		2.00	912.16667	1440.26354	.534	-2102.3396	3926.6729	
		3.00	46.53333	1510.56114	.976	-3115.1075	3208.1741	
Time30min	1.00	2.00	1024.50000	1751.64436	.566	-2641.7338	4690.7338	
		3.00	-548.43333	1837.14010	.769	-4393.6118	3296.7451	
		4.00	-285.83333	1751.64436	.872	-3952.0671	3380.4004	
	2.00	1.00	-1024.50000	1751.64436	.566	-4690.7338	2641.7338	
		3.00	-1572.93333	1837.14010	.403	-5418.1118	2272.2451	
		4.00	-1310.33333	1751.64436	.464	-4976.5671	2355.9004	
	3.00	1.00	548.43333	1837.14010	.769	-3296.7451	4393.6118	
		2.00	1572.93333	1837.14010	.403	-2272.2451	5418.1118	
		4.00	262.60000	1837.14010	.888	-3582.5784	4107.7784	
	4.00	1.00	285.83333	1751.64436	.872	-3380.4004	3952.0671	
		2.00	1310.33333	1751.64436	.464	-2355.9004	4976.5671	
		3.00	-262.60000	1837.14010	.888	-4107.7784	3582.5784	
Time60min	1.00	2.00	3070.50000	1700.49592	.087	-488.6789	6629.6789	
		3.00	1727.33333	1783.49517	.345	-2005.5649	5460.2316	
		4.00	2455.16667	1700.49592	.165	-1104.0122	6014.3455	
	2.00	1.00	-3070.50000	1700.49592	.087	-6629.6789	488.6789	
		3.00	-1343.16667	1783.49517	.461	-5076.0649	2389.7316	
		4.00	-615.33333	1700.49592	.721	-4174.5122	2943.8455	
	3.00	1.00	-1727.33333	1783.49517	.345	-5460.2316	2005.5649	
		2.00	1343.16667	1783.49517	.461	-2389.7316	5076.0649	
		4.00	727.83333	1783.49517	.688	-3005.0649	4460.7316	
	4.00	1.00	-2455.16667	1700.49592	.165	-6014.3455	1104.0122	
		2.00	615.33333	1700.49592	.721	-2943.8455	4174.5122	
		3.00	-727.83333	1783.49517	.688	-4460.7316	3005.0649	
Time70min	1.00	2.00	5130.83333*	1459.04379	.002	2077.0196	8184.6471	
		3.00	2823.66667	1530.25803	.081	-379.2002	6026.5335	
		4.00	1675.66667	1459.04379	.265	-1378.1471	4729.4804	
	2.00	1.00	-5130.83333*	1459.04379	.002	-8184.6471	-2077.0196	
		3.00	-2307.16667	1530.25803	.148	-5510.0335	895.7002	
		4.00	-3455.16667*	1459.04379	.029	-6508.9804	-401.3529	
	3.00	1.00	-2823.66667	1530.25803	.081	-6026.5335	379.2002	
		2.00	2307.16667	1530.25803	.148	-895.7002	5510.0335	
		4.00	-1148.00000	1530.25803	.462	-4350.8669	2054.8669	
	4.00	1.00	-1675.66667	1459.04379	.265	-4729.4804	1378.1471	
		2.00	3455.16667*	1459.04379	.029	401.3529	6508.9804	
		3.00	1148.00000	1530.25803	.462	-2054.8669	4350.8669	
Time80min	1.00	2.00	4309.83333*	1440.94478	.008	1293.9012	7325.7654	
		3.00	2914.43333	1511.27564	.069	-248.7029	6077.5696	
		4.00	1853.83333	1440.94478	.214	-1162.0988	4869.7654	
	2.00	1.00	-4309.83333*	1440.94478	.008	-7325.7654	-1293.9012	
		3.00	-1395.40000	1511.27564	.367	-4558.5363	1767.7363	
		4.00	-2456.00000	1440.94478	.105	-5471.9321	559.9321	
	3.00	1.00	-2914.43333	1511.27564	.069	-6077.5696	248.7029	
		2.00	1395.40000	1511.27564	.367	-1767.7363	4558.5363	
		4.00	-1060.60000	1511.27564	.491	-4223.7363	2102.5363	
	4.00	1.00	-1853.83333	1440.94478	.214	-4869.7654	1162.0988	
		2.00	2456.00000	1440.94478	.105	-559.9321	5471.9321	
		3.00	1060.60000	1511.27564	.491	-2102.5363	4223.7363	
Time90min	1.00	2.00	5874.00000*	1318.88134	.000	3113.5496	8634.4504	
		3.00	4764.33333*	1383.25442	.003	1869.1486	7659.5181	
		4.00	2998.50000*	1318.88134	.035	238.0496	5758.9504	
	2.00	1.00	-5874.00000*	1318.88134	.000	-8634.4504	-3113.5496	
		3.00	-1109.66667	1383.25442	.432	-4004.8514	1785.5181	
		4.00	-2875.50000*	1318.88134	.042	-5635.9504	-115.0496	
	3.00	1.00	-4764.33333*	1383.25442	.003	-7659.5181	-1869.1486	
		2.00	1109.66667	1383.25442	.432	-1785.5181	4004.8514	
		4.00	-1765.83333	1383.25442	.217	-4661.0181	1129.3514	
	4.00	1.00	-2998.50000*	1318.88134	.035	-5758.9504	-238.0496	
		2.00	2875.50000*	1318.88134	.042	115.0496	5635.9504	
		3.00	1765.83333	1383.25442	.217	-1129.3514	4661.0181	
Time100min	1.00	2.00	6142.66667*	1202.31366	.000	3626.1952	8659.1381	
		3.00	4237.33333*	1260.99721	.003	1598.0358	6876.6308	
		4.00	2979.83333*	1202.31366	.023	463.3619	5496.3048	
	2.00	1.00	-6142.66667*	1202.31366	.000	-8659.1381	-3626.1952	
		3.00	-1905.33333	1260.99721	.147	-4544.6308	733.9642	
		4.00	-3162.83333*	1202.31366	.016	-5679.3048	-646.3619	
	3.00	1.00	-4237.33333*	1260.99721	.003	-6876.6308	-1598.0358	
		2.00	1905.33333	1260.99721	.147	-733.9642	4544.6308	
		4.00	-1257.50000	1260.99721	.331	-3896.7975	1381.7975	
	4.00	1.00	-2979.83333*	1202.31366	.023	-5496.3048	-463.3619	
		2.00	3162.83333*	1202.31366	.016	646.3619	5679.3048	
		3.00	1257.50000	1260.99721	.331	-1381.7975	3896.7975	
Time110min	1.00	2.00	6590.50000*	1320.67822	.000	3826.2887	9354.7113	
		3.00	5120.26667*	1385.13901	.002	2221.1374	8019.3959	
		4.00	3470.66667*	1320.67822	.017	706.4554	6234.8780	
	2.00	1.00	-6590.50000*	1320.67822	.000	-9354.7113	-3826.2887	
		3.00	-1470.23333	1385.13901	.302	-4369.3626	1428.8959	
		4.00	-3119.83333*	1320.67822	.029	-5884.0446	-355.6220	
	3.00	1.00	-5120.26667*	1385.13901	.002	-8019.3959	-2221.1374	
		2.00	1470.23333	1385.13901	.302	-1428.8959	4369.3626	
		4.00	-1649.60000	1385.13901	.248	-4548.7293	1249.5293	
	4.00	1.00	-3470.66667*	1320.67822	.017	-6234.8780	-706.4554	
		2.00	3119.83333*	1320.67822	.029	355.6220	5884.0446	
		3.00	1649.60000	1385.13901	.248	-1249.5293	4548.7293	
Time120min	1.00	2.00	7143.83333*	1390.74908	.000	4232.9621	10054.7046	
		3.00	5068.93333*	1458.62994	.003	2015.9858	8121.8809	
		4.00	2492.83333	1390.74908	.089	-418.0379	5403.7046	
	2.00	1.00	-7143.83333*	1390.74908	.000	-10054.7046	-4232.9621	
		3.00	-2074.90000	1458.62994	.171	-5127.8476	978.0476	
		4.00	-4651.00000*	1390.74908	.003	-7561.8713	-1740.1287	
	3.00	1.00	-5068.93333*	1458.62994	.003	-8121.8809	-2015.9858	
		2.00	2074.90000	1458.62994	.171	-978.0476	5127.8476	
		4.00	-2576.10000	1458.62994	.093	-5629.0476	476.8476	
	4.00	1.00	-2492.83333	1390.74908	.089	-5403.7046	418.0379	
		2.00	4651.00000*	1390.74908	.003	1740.1287	7561.8713	
		3.00	2576.10000	1458.62994	.093	-476.8476	5629.0476	

*. §¡­È®tªº显µÛ©Ê¤ô¥­为 0.05¡C	
