# Supplementary material for: Taxifolin protects rat against myocardial ischemia/reperfusion injury by modulating the mitochondrial apoptosis pathway
Source: PeerJ. 2019 Jan 31;7:e6383. doi: 10.7717/peerj.6383 (PMC6360081; doi:10.7717/peerj.6383)
Supplement: Supplemental Information 6 [file peerj-07-6383-s006.zip › Statistical Reporting/Analysis results/Word file form/SOD.doc]

ONEWAY SOD BY Group
  /STATISTICS HOMOGENEITY
  /MISSING ANALYSIS
  /POSTHOC=LSD ALPHA(0.05).

Oneway

C:\Users\Administrator\Desktop\Statistical Reporting\SOD.sav

Test of Homogeneity of Variances	
SOD  	
Levene Statistic	df1	df2	Sig.	
1.576	3	22	.224	

ANOVA	
SOD  	
	Sun of Squares	df	Mean Square	F	Sig.	
Between Groups	16156.361	3	5385.454	18.725	.000	
Within Groups	6327.421	22	287.610			
Total	22483.783	25				

Post Hoc Tests
Multiple Comparisons	
Dependent Variable: SOD  	
LSD  	
(I) Group	(J) Group	Mean Difference (I-J)	Std. Error	Sig.	95% Confidence interval	
					Lower Bound	Lower Bound	
1.00	2.00	65.92363*	9.15894	.000	46.9291	84.9181	
	3.00	20.07545	10.26923	.063	-1.2216	41.3725	
	4.00	37.79100*	9.43516	.001	18.2237	57.3583	
2.00	1.00	-65.92363*	9.15894	.000	-84.9181	-46.9291	
	3.00	-45.84818*	9.66816	.000	-65.8987	-25.7976	
	4.00	-28.13263*	8.77715	.004	-46.3353	-9.9299	
3.00	1.00	-20.07545	10.26923	.063	-41.3725	1.2216	
	2.00	45.84818*	9.66816	.000	25.7976	65.8987	
	4.00	17.71554	9.93021	.088	-2.8785	38.3095	
4.00	1.00	-37.79100*	9.43516	.001	-57.3583	-18.2237	
	2.00	28.13263*	8.77715	.004	9.9299	46.3353	
	3.00	-17.71554	9.93021	.088	-38.3095	2.8785	

*. The mean difference is significant at the 0.05 level.	
